# Genetically Engineered IL12/CSF1R‐Macrophage Membrane‐Liposome Hybrid Nanovesicles for NIR‐II Fluorescence Imaging‐Guided and Membrane‐Targeted Mild Photothermal‐Immunotherapy of Glioblastoma

**DOI:** 10.1002/advs.202500131

**Published:** 2025-04-25

**Authors:** Pengfei Chen, Yue Liu, Haiyan Huang, Menglong Li, Hui Xie, Shubham Roy, Jingsi Gu, Jian Jin, Kai Deng, Lixin Du, Bing Guo

**Affiliations:** ^1^ Department of Traumatic Orthopedics Shenzhen Longhua District Central Hospital Shenzhen 518110 China; ^2^ School of Science Shenzhen Key Laboratory of Advanced Functional Carbon Materials Research and Comprehensive Application Harbin Institute of Technology Shenzhen 518055 China; ^3^ Chengdu Institute of Organic Chemistry Chinese Academy of Sciences Chengdu 610041 China; ^4^ Education Center and Experiments and Innovations Harbin Institute of Technology Shenzhen 518055 China; ^5^ Department of Medical Imaging Shenzhen Longhua District Key Laboratory of Neuroimaging Shenzhen Longhua District Central Hospital Shenzhen 518110 China

**Keywords:** biomimetic strategy, glioblastoma, immunotherapy, NIR‐II fluorescence imaging, photothermal therapy

## Abstract

It is a big challenge for precision therapy of glioblastoma, mainly due to the existence of blood–brain barrier (BBB), tumor immunosuppressive microenvironment (TIM), and lack of efficient treatment paradigms. Herein, a theranostic nanoplatform for the second near‐infrared window (NIR‐II) fluorescence imaging‐guided membrane‐targeted mild photothermal‐immunotherapy of glioblastoma using genetically engineered CSF1R/IL12‐macrophage membrane (MM)‐liposome hybrid nanovesicles, is reported. By mimicking lipophilic membrane probe (Dil) with octadecyl chains, a NIR‐II emissive photothermal dye (**IRC18**), which realizes labeling of nanovesicle lipid bilayers for biodistribution tracing, glioblastoma diagnosis, and molecular imaging of tumoral microenvironment, is synthesized. Importantly, MM and c‐RGD‐decorated liposome together offer BBB crossing, tumor targeting, and long‐term circulation; while, the genetically overexpressed CSF1R and IL12 on MM surface contribute to effective modulation of M2‐to‐M1 macrophage repolarization and local promotion of T cell cytotoxicity in glioblastoma microenvironment, respectively. Notably, through membrane fusion, **IRC18** dyes translocate from nanovesicle lipid bilayers to glioblastoma membranes, which achieve membrane‐targeted mild photothermal therapy to ablate primary tumor and induce immunogenic cell death to promote antigen presentation. More importantly, the combined blockade of the CSF1‐CSF1R axis and IL‐12 enrichment not only reprograms the tumor microenvironment through macrophage M1 repolarization but also activates cytotoxic T cells, ultimately achieving complete glioblastoma eradication. This research provides an efficient theranostic paradigm for glioblastoma treatment.

## Graphical abstract

A theranostic nanoplatform is formulated with NIR‐II emissive lipophilic cyanine dye IRC18 to intercalate into genetically engineered IL12/CSF1R‐macrophage membrane‐liposome hybrid nanovesicles, which assists to achieve blood–brain barrier crossing, glioblastoma targeting, NIR‐II fluorescence imaging, image‐guided membrane‐targeted mild photothermal therapy to ablate primary brain tumors, and modulation of tumor immunosuppressive microenvironment via CSF‐1‐CSF1R axis blocking and IL12 enrichment for boosted immunotherapy.

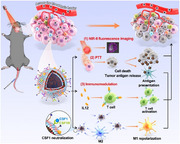

## 1 Introduction

Glioblastoma (GBM) is one of the most lethal diseases with rapid progression and high invasiveness.^[^ ^1^, ^2^, ^3^, ^4^ ^]^ An approach of surgery combined with radiotherapy and chemotherapy has become the state‐of‐the‐art therapeutic paradigm, but patients often suffer from postoperative neurological impairment or recurrence with a relatively low average median survival lifespan (<2 years). Researchers have been devoted to exploring new drug carriers and corresponding therapeutic paradigms such as gene therapy, photothermal therapy (PTT), photodynamic therapy (PDT), chemodynamic therapy (CDT), and immunotherapies.^[^ ^5^, ^6^, ^7^, ^8^, ^9^ ^]^ However, these treatment modalities still show poor prognosis for patients, which is mainly due to limiting factors including drug resistance, tumor heterogeneity and invasiveness, blood–brain barrier (BBB), and tumor immunosuppressive microenvironment (TIM).^[^ ^7^, ^10^, ^11^ ^]^ Thus, novel precision therapy platforms to circumvent these limiting factors are urgently needed for GBM treatment.

Combinatory therapy as an emerging and promising treatment paradigm for cancer therapy could synergistically integrate multiple merits of each modality, overcome the shortcomings of individual therapeutics, and lower the threshold of therapeutic drug dosage with diminished side‐effects.^[^ ^12^, ^13^, ^14^, ^15^ ^]^ Recently, combinatory immunotherapy and other therapeutics (e.g. chemo, radio, PTT, PDT, and CDT) with self‐cascading penetrative characteristics have shown improved efficacy in GBM ablation.^[^ ^16^, ^17^, ^18^, ^19^, ^20^ ^]^ The multiscale mechanisms of drug action generally include i) BBB crossing and targeting to brain tumors for therapeutic agents; ii) direct eradication of primary tumors via therapy; iii) therapy‐induced immunogenic cell death (ICD) to promote antigen presentation; iv) activation of antigen‐presenting cells (APCs); v) proliferation and infiltration of immune cells into the tumor microenvironment to eliminate residual, disseminated, and even metastatic tumors beyond therapy scope and prevent tumor recurrence; and vi) immunologic memory to prevent tumor recurrence.^[^ ^16^, ^17^, ^18^, ^19^, ^20^ ^]^

PTT is efficient in locally killing tumor cells with good spatiotemporal controllability and low incidence of drug resistance.^[^ ^21^, ^22^, ^23^, ^24^ ^]^ As known, hyperthermia could induce ICD and trigger the release of tumor‐associated antigens (TAAs) and damage‐associated molecular patterns (DAMPs) from ablated tumor cell residues, which are required for activating proinflammatory immune responses to provoke antitumor immunity.^[^ ^25^ ^]^ However, there also exist limiting factors in PTT as follows: i) Normally the hyperthermia generated in the PTT process should reach above 50 °C to destroy the tumor structures owing to the limited light penetration depth.^[^ ^23^, ^24^, ^25^, ^26^ ^]^ However, this high temperature would bring in unwanted damage to neighboring normal tissues, which is a big concern, especially for the treatment of GBM surrounded with important neurological tissues. Thus, mild PTT would be a good alternative to minimize unwanted damage,^[^ ^27^, ^28^ ^]^ but it might be at the expense of therapeutic efficacy because of insufficient heat damage. ii) The immune response caused by the immune‐associated tumor antigens yielded via PTT treatment alone is relatively weak and cannot completely suppress tumor recurrence and metastasis,^[^ ^27^, ^28^ ^]^ especially in “cold”/immunosuppressive GBM microenvironment which can dramatically downregulate antitumor immune response.^[^ ^29^, ^30^ ^]^ Recently, targeting therapy of subcellular organelles (e.g. membrane, mitochondria, and nucleus) with small molecules or nanomaterials has been demonstrated with the capability to precisely and locally trigger organelle‐mediated cell death signals, boost eradication efficiency of tumors, and reduce side effects.^[^ ^31^, ^32^, ^33^ ^]^ Especially, for the cell membrane‐targeted therapy, it exhibits high antitumor efficacy by avoiding cellular barriers and drug efflux.^[^ ^34^, ^35^ ^]^ Thus, it is anticipated that cell membrane‐targeted mild PTT, in combination with immunotherapy, is capable of circumventing the limiting factors of GBM treatment and achieving the goals of eradicating primary tumors, generating enhanced antitumor immune responses, preventing tumor recurrence and metastasis, which has rarely been reported so far.

Modulation of TIM is crucial for efficient cancer immunotherapy.^[^ ^34^ ^]^ Importantly, tumor‐associated macrophages (TAMs) are the prominent component in TIM. For macrophages and glioma cells, there exist reciprocal effects on the survival, proliferation, or polarization of each other by promoting tumorigenesis, invasiveness, and metastasis. In addition, TAMs play an important role in restraining adaptive immune response through inducing dysfunction of dendritic cells (DCs) and tumor‐infiltrating effector T cells, and this severely limits immunotherapeutic efficacy. As macrophage colony‐stimulating factor (MCSF, also known as CSF1) is abundantly secreted by tumor cells, ligation of CSF1 to the colony‐stimulating factor 1 receptor (CSF1R) on the macrophage membrane (MM) not only facilitates the differentiation, proliferation, and survival of monocytes/macrophages but also promotes the polarization of tumor‐supportive M2 phenotype.^[^ ^36^ ^]^ Therefore, blocking the core CSF1‐CSF1R signal axis could abrogate macrophage‐glioma cell heterotypic signaling, inhibit the polarization of immunosuppressive M2 macrophage in the TIM, and facilitate recruiting tumor‐infiltrating T cells.^[^ ^37^ ^]^ So far, monoclonal antibodies or small molecule inhibitors targeting the CSF1‐CSF1R axis are being used in clinical trials but have some limitations due to inefficacy or drug resistance.^[^ ^38^, ^39^ ^]^ For instance, Chen et al. demonstrated that TAM membrane‐coated nanovesicles were capable of binding CSF1 in vitro and in vivo through CSF1R expressed on the TAM membrane and promoted M1 macrophage polarization.^[^ ^40^ ^]^ These results indicate that CSF1R‐expressing membrane‐coated nanovesicles can act as an ideal decoy sponge to neutralize CSF1; thus, inhibiting protumor M2 macrophage polarization.

Notably, Interleukin 12 (IL12), a heterodimeric pro‐inflammatory cytokine, also plays a critical role in modulating the antitumor immune responses. It is especially critical for T helper 1 (Th1) cell differentiation and antibody production.^[^ ^41^ ^]^ Importantly, IL12 stimulates T cells and natural killer cells to exert effector functions through induction of cytotoxic enzymes and cytokines, mainly interferon‐γ (IFN‐γ), and further inhibits or reprograms immunosuppressive cells, such as intratumor T regulatory cells, TAMs, and myeloid‐derived suppressor cells (MDSCs); thus, converting immunologically “cold” tumors into “hot” ones.^[^ ^41^ ^]^ Recently, recombinant mouse IL12 (active proteins or IL12 nucleic acid drugs) has been used as a key “chaperone agent” to elicit potent antitumor effects in numerous mouse models, inducing fast generation of antitumor T cell immune responses and tumor suppression.^[^ ^42^, ^43^ ^]^ Taken together, it is anticipated that dual CSF1‐CSF1R axis blocking and IL12 administration together would reverse immunosuppressive tumor microenvironment and boost therapeutic outcomes.

As known, macrophages possess inherent tumor‐homing capabilities as they can cross multiple biological barriers such as the dense tumor extracellular matrix, tight vascular wall, or BBB via responding to various chemoattractants and hypoxic conditions in the tumor microenvironment. Researchers developed “Trojan horse”‐mimic strategy using engineered macrophages and macrophage‐derived components (e.g., MM, extracellular vesicles, and exosomes) as vectors to physically or chemically encapsulate imaging and therapy drugs and deliver them to brain tumors.^[^ ^44^ ^]^ This biomimetic strategy has shown outstanding merits in high efficiency in BBB crossing, long‐term circulation, and intrinsic targeting capability to brain tumors. Recently, Dai et al. showed that NIR‐II fluorescence imaging could assist to real‐time molecularly evaluate the tumor microenvironment in response to immunotherapy and targeting capabilities for the nanoplatforms in vivo, which outperform the conventional visible/NIR‐I fluorescence imaging.^[^ ^45^ ^]^ With improved fidelity in penetration depth and spatiotemporal resolution, NIR‐II fluorescence imaging also showed potential in GBM diagnosis and image‐guided therapy.^[^ ^46^, ^47^ ^]^ Thus, it is reasonably hypothesized that NIR‐II fluorescence imaging‐guided PTT, in combination with immunotherapy by biomimetic MM‐coated nanovesicles as carriers, would contribute to efficient GBM treatment.

**Scheme 1:**
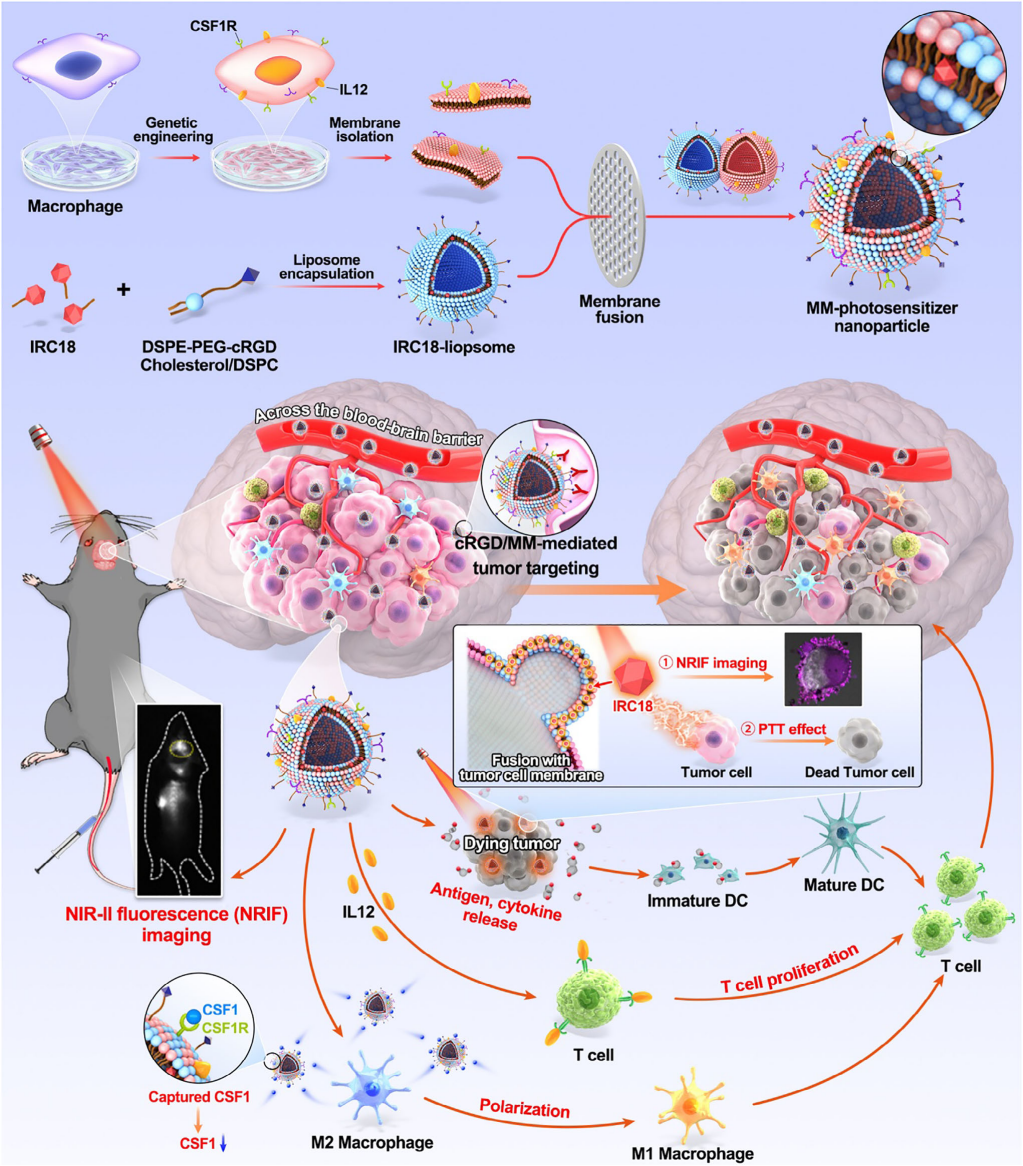
A biomimetic theranostic nanoplatform with genetically engineered CSF1R/IL12‐MM‐liposome hybrid nanovesicles containing NIR‐II fluorescence dye (**IRC18**) for NIR‐II fluorescence imaging‐guided and membrane‐targeted mild photothermal‐immunotherapy of GBM.

It should be noticed that systemic administration of immune drugs (such as cytokine, antibodies, and inhibitors) and other therapeutic drugs generally show limited efficacy^[^ ^40^ ^]^ because of their short half‐life in circulation and the lethal on‐target and off‐tumor side effects.^[^ ^48^ ^]^ The compact combination of the therapeutic and imaging agents together in a single biomimetic MM‐coated nanoplatform would exhibit pharmacokinetic advantages to minimize these limitations.^[^ ^49^, ^50^ ^]^ In this contribution, we used a genetic engineering method to decorate both CSF1R and IL12 on MM for combinatory immunotherapeutic effects, and formulated hybrid nanovesicles with the MM and c‐RGD‐decorated PEGylated liposomes,^[^ ^23^, ^47^, ^51^ ^]^ which were integrated with lipophilic NIR‐II emissive photothermal dye (**IRC18**) for NIR‐II fluorescence imaging‐guided mild photothermal‐immunotherapy of GBM (**Scheme** **1**). The CSF1R/IL12‐MM‐liposome hybrid nanovesicles would inherit properties from source materials including BBB crossing, GBM targeting, immunomodulatory effects, and long‐term circulation and colloidal stability. The MM‐liposome hybrid nanovesicles assist **IRC18** with a balanced lipophilic backbone and flexible alkyl chains to fuse into tumor cell membrane bilayer. Thus, this would realize locally subcellular membrane‐targeted mild hyperthermia under near‐infrared laser irradiation to effectively destruct primary GBM cells, followed by PTT‐initiated ICD. Simultaneously, synergistic blocking of CSF1‐CSF1R axis and eliciting of IL12‐induced T cell cytotoxicity would further eradicate the GBM locally and in distance. In this study, we illustrated the formulation process of **IRC18** and hybrid nanovesicles at the beginning and further characterized the physical, chemical, and biological performance (e.g., uptake, light/dark cytotoxicity, combinatory photothermal and immunotherapy) of the CSF1R/IL12‐MM‐IRC18‐liposome hybrid nanovesicles (CSF1R/IL12‐MM‐IRC18‐LPS) in vitro and in vivo.

## 2 Results and Discussion

### 2.1 Synthesis and Characterization of Nanoplatform

#### 2.1.1 Synthesis and Characterization of Dye **IRC18**

**Figure 1:**
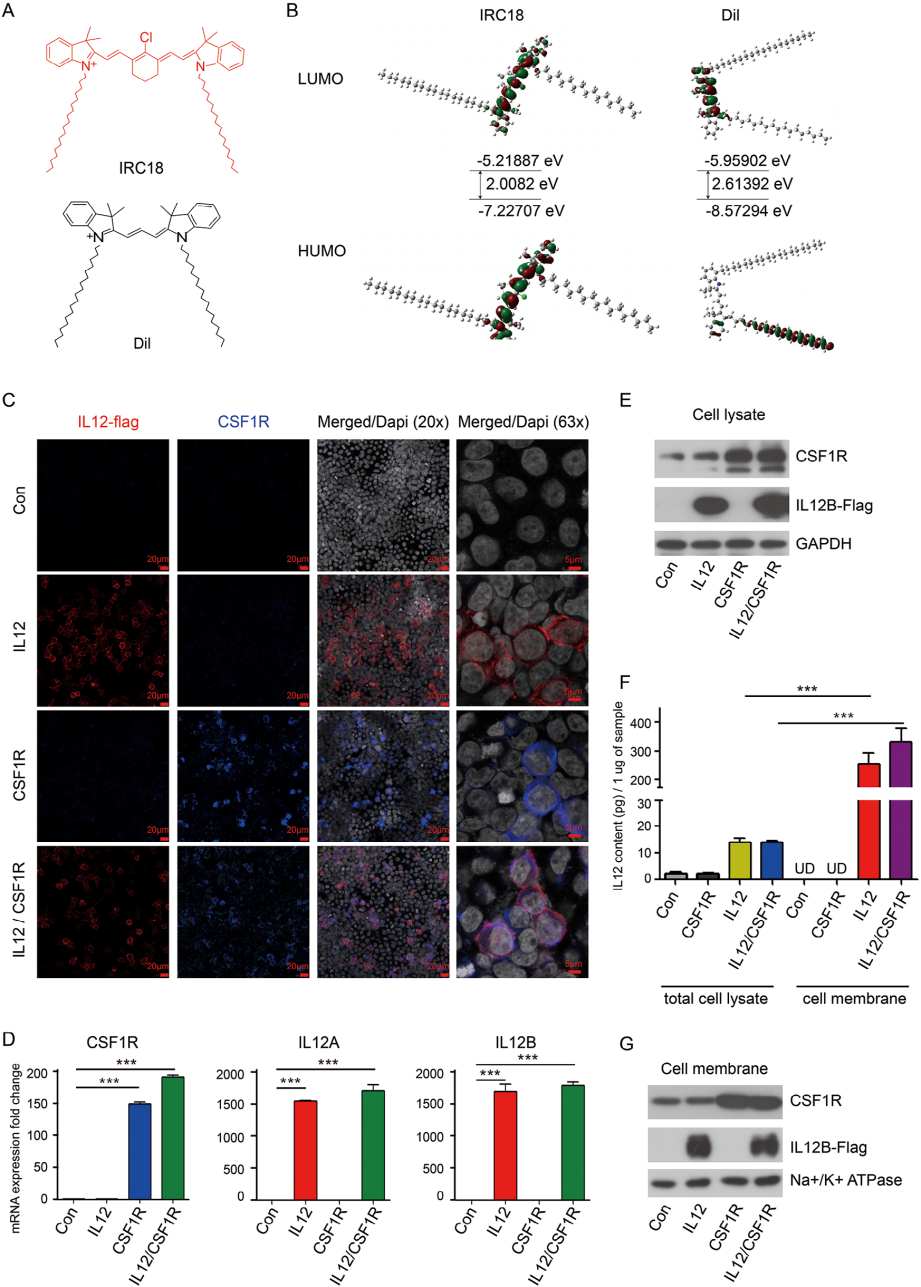
A) Chemical structure of IRC18 and Dil; B) HOMO and LUMO of IRC18; C) immunofluorescence staining of CSF1R and IL12 in 293T cells overexpressing CSF1R or IL12 gene cassette; D) quantitative mRNA expression of CSF1R, IL12A, and IL12B in Raw264.7 cells stably overexpressing CSF1R or IL12 gene cassette; E) immunoblot of CSF1R and IL12B in Raw264.7 cells stably overexpressing CSF1R or IL12 gene cassette; F) the protein levels of IL12 in total cell lysate and isolated membranes from Raw264.7 cells stably overexpressing CSF1R or IL12 gene cassette was measured by ELISA; and G) immunoblot of CSF1R and IL12B in isolated membranes. ***p < 0.001 by Student's *t‐*test.

Lipophilic cyanine derivatives such as Dil, with absorption and emission in the visible range, have been developed for labeling of membrane of living cells and even the bilayer membrane of biomimetic nanovesicles for in vivo fluorescence tracking.^[^ ^52^ ^]^ As known, as compared to conventional visible and NIR‐I fluorescence imaging, NIR‐II fluorescence imaging exhibits significant improvement in imaging fidelity and depth, spatiotemporal resolution, and signal/background ratio. In addition, NIR‐II fluorescence imaging agents intrinsically exhibit strong NIR absorbance with potential for PTT applications.^[^ ^53^ ^]^ Thus, we synthesized a NIR‐II emissive dye **IRC18** via the Knoevenagel reaction (Scheme S1, Supporting Information), by mimicking the lipophilic Dil with octadecyl chain and extending the conjugation length (**Figure** **1**A), which could be used as membrane labeling dyes for in vitro and in vivo tracing and formulation of MM‐liposome hybrid nanovesicles for mild photothermal and immunotherapy of GBM.

Dil exhibits absorption and emission at 554 and 571 nm in DMSO, respectively, whereas **IRC18** shows absorption and emission at 796 and 908 nm in the same conditions (Figure S1A,B, Supporting Information). In PBS solution, Dil exhibits absorption and emission at 556 and 669 nm, respectively. Under the same conditions, **IRC18** shows absorption and emission at 796 and 996 nm, respectively (Figure S1C,D, Supporting Information). This indicates that the conjugation length extension effectively red‐shifts the absorption and emission. Further, the DFT calculation shows that the bandgap for **IRC18** is much narrower than Dil (Figure 1B), which goes well with the fact that **IRC18** exhibited red‐shited absorbance and emission as compared to that of Dil (Figure S1, Supporting Information).

#### 2.1.2 Genetic Engineering of MMs

Recently, the consolidation of biomimetic nanoplatforms with macrophage membranes has received increasing interest, owing to their natural tumor‐homing capabilities, which enable targeted delivery of internalized drugs, crossing of the tumor core or BBB through chemotactic aptitude, prolonged systemic circulation time, and delayed clearance by the reticuloendothelial system.^[^ ^54^ ^]^ To leverage the advantages of macrophages; while, minimizing the systemic cytotoxicity of free immune drugs, we proposed the construction of genetically engineered macrophage membrane‐based biomimetic nanoplatforms. These nanoplatforms consist of CSF1R and IL12 proteins on the surface of macrophage membranes and the photothermal agent **IRC18** in the liposomal bilayer. Given that the CSF1R protein acts as an ideal decoy sponge to neutralize CSF1 signaling and IL12 serves as a strong agonist for T cell activation, we hypothesize that the combination of M2‐to‐M1 macrophage repolarization via CSF1R and tumor‐specific delivery of IL12 to activate anti‐tumor T cell responses would de novo reprogram the tumor microenvironment for efficient tumor elimination.^[^ ^50^, ^55^ ^]^ To equip macrophage membranes with these dual functions, a macrophage cell line Raw264.7 was selected for genetical engineering with stable overexpression of CSF1R and IL12 on the cell membrane. As known, IL12 is a secreted cytokine which is composed of two polypeptide chains, an *α* chain p35 and a *β* chain p40 subunit. To enable membrane expression of IL12 via a glycosylphosphatidylinositol (GPI) anchor, we designed a gene expression cassette comprising an IL12A‐P2A sequence fragment, followed by an amino‐terminal signal sequence for membrane translocation into the endoplasmic reticulum (ER), a LQEFAT linker, IL12B, 3× Flag sequence, a LEN linker, and a carboxyl‐terminal GPI anchor signal sequence derived from the Thy‐1 gene.^[^ ^56^ ^]^ Using immunofluorescence staining, we first verified the cell membrane localization of both CSF1R and IL12 by expressing their respective gene expression cassettes in 293T cells (Figure 1C). Quantitative polymerase chain reaction (qPCR) and western blotting results confirmed the successful overexpression of both CSF1R and IL12 in the engineered macrophages (Figure 1D,E). Subsequently, the cell membranes were isolated from these cells. The IL12 concentration was quantified using enzyme‐linked immunosorbent assay (ELISA). Approximately 300 pg per µg of cell membrane component was obtained from the engineered macrophages (Figure 1F). In addition, western blotting confirmed the successful overexpression of both CSF1R and IL12 in the cell membrane component (Figure 1G). The membranes from these engineered cells were then collected and coated onto a nanoparticulate substrate for in vitro and in vivo imaging and therapy.

#### 2.1.3 Synthesis and Characterization of CSF1R/IL12‐MM‐Liposome Hybrid Nanovesicles Containing **IRC18** (IL12/CSF1R‐MM‐IRC18‐LPS)

It is noticed that the large conjugation structure makes **IRC18** with poor water solubility, which inhibits the direct use of **IRC18** water solution to stain cell membranes. To address this problem, we inserted **IRC18** in the lipid bilayer of liposomes at the beginning, which could be decorated into MM‐liposome hybrid nanovesicles during squeezing process and further intercalated into GBM cell membrane through the fusion between the lipid bilayer of membrane‐liposome hybrid nanovesicles and GBM cell membranes. At the beginning, IL12/CSF1R‐MMs were purified (Figure S2, Supporting Information) and IRC18 liposome (IRC18‐LPS) was generated by lipid film hydration and extrusion to construct **IRC18** containing liposomes (Scheme 1). Finally, membrane extrusion was applied to facilitate the fusion of lipid bilayers between IRC18‐LPS and IL12/CSF1R‐MMs (IL12/CSF1R‐MM) with a ratio of 1 mg protein equivalent of membranes and 1 mg of lipid film, yielding **IRC18‐**containing IL12/CSF1R‐MM‐liposome hybrid nanovesicles (IL12/CSF1R‐MM‐IRC18‐LPS) (Scheme 1).

**Figure 2:**
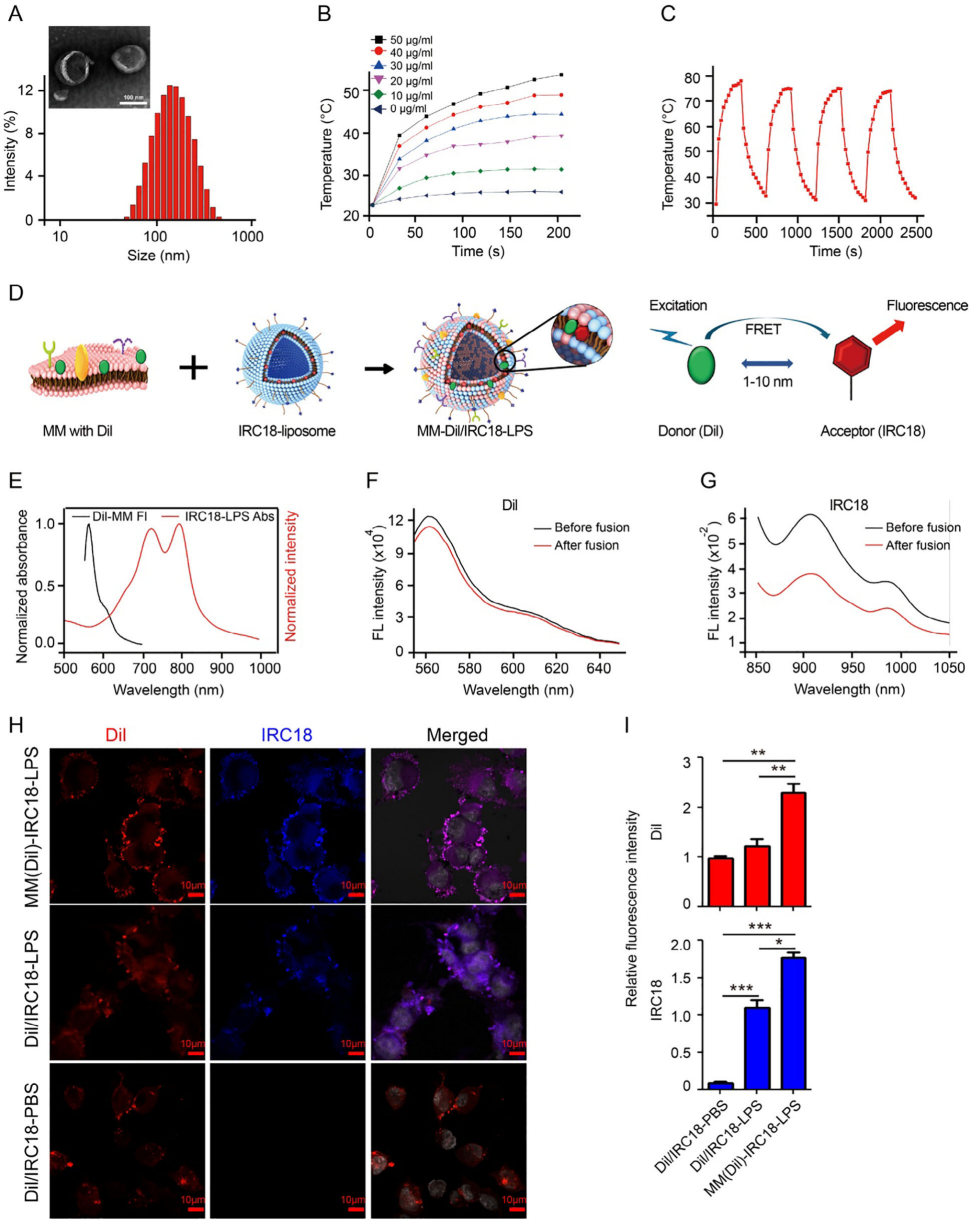
A) Morphology of IL12/CSF1R‐MM‐IRC18‐LPS nanovesicles and particle size distribution in IL12/CSF1R‐MM‐IRC18‐LPS, as tested by transmission electron microscopy. B) Temperature profiles of IL12/CSF1R‐MM‐IRC18‐LPS at different concentrations under laser irradiation at 808 nm wavelength and photon energy of 1 W cm^−2^. C) Temperature profiles of IL12/CSF1R‐MM‐IRC18‐LPS under four cycles of laser irradiation at 808 nm wavelength with photon energy of 1 W cm^−2^. Temperature profiles of IL12/CSF1R‐MM‐IRC18‐LPS nanovesicles under four cycles of laser irradiation at 808 nm with a photon energy of 1 W cm^−2^. D) Schematic illustration of the FRET effect occurring in the Dil‐stained endogenous drug carriers and liposomes made of IRC18. E) The emission profiles of the Dil‐MM versus the absorption curves of IRC18‐LPS. F) Fluorescence changes of Dil before and after freeze‐thawing. G) Fluorescence changes of IRC18 before and after freeze‐thawing. H) Confocal microscopy imaging of GL261 cells treated with MM (DIL)‐IRC18‐LPS, DIL/IRC18‐LPS, and DIL/IRC18‐PBS for 2 h. I) Statistics of the relative fluorescence intensity of Dil and IRC18 in cells as indicated in (H). *p < 0.05, **p < 0.01, and ***p < 0.001 by Student's *t*‐test.

The IL12/CSF1R‐MM‐IRC18‐LPS sample exhibited spherical morphology with diameters of 112 and 139 nm by TEM and DLS, respectively (**Figure** **2**A). Further, for IL12/CSF1R‐MM‐IRC18‐LPS, the NIR‐II fluorescence quantum yield was characterized to be 1.78% with reference to IR1061, a typical IR‐II dye with a NIR‐II quantum yield of 0.75% (Figure S3, Supporting Information). The spherical morphology of hybrid vesicles indicates that MMs exhibited reduced mechanical stiffness during membrane fusion. It was found that IL12/CSF1R‐MM‐IRC18‐LPS could maintain colloidal stability in aqueous conditions for 4 weeks in different media including PBS, H2O, FBS, and DMEM (Figure S4A, Supporting Information). It is mainly attributed to the liposomal PEG on the nanovesicle surface to inhibit particle aggregation.^[^ ^57^ ^]^ The size and polydispersity (PDI) of hybrid nanovesicles are located in the middle between their mother sources including liposomes (IRC18‐LPS) and MM (IL12/CSF1R‐MM) (Figure S4B,C, Supporting Information), suggesting the particle fusion during the hybrid formulation process. The zeta potential value of IL12/CSF1R‐MM‐IRC18‐LPS was intermediate between that of IL12/CSF1R‐MM (abbreviated as MM) and IRC18‐LPS, which further indicated that the MMs were successfully fused with the artificial lipid membrane (Figure S4D, Supporting Information).

The photothermal effect was further investigated by varying the dye concentration of aqueous samples. The hyperthermia temperature changes under continuous 808 nm laser irradiation (1 W cm^−2^) were monitored with an infrared (IR) thermal imaging camera (Figure 2B,C; Figure S5, Supporting Information). It was noticed that the PTT temperature grew quickly with either the increasing laser irradiation time or the increasing sample concentration (Figure 2B). For example, the aqueous sample (50 µg mL^−1^) exhibited a sharp temperature increase to ≈54 °C within 5 min (Figure 2B), which suggests excellent photothermal conversion capability. Further, the photothermal conversion efficiency for the sample was calculated to be 71.4% (Figure S5, Supporting Information), which was much higher than most reported photothermal agents.^[^ ^58^ ^]^ Moreover, the aqueous sample exhibited negligible change in reversible photothermal heating and cooling for four runs (Figure 2C). These results indicate that the membrane‐liposome hybrid nanoplatform exhibited good photothermal conversion and stability, which is valuable for long‐term and repeated PTT treatment of cancers.

Fluorescence resonance energy transfer (FRET) study was applied to evaluate the fusion between IL12/CSF1R‐MM and the IRC18‐LPS.^[^ ^59^ ^]^ As is known, FRET is a non‐radiative near‐field resonance phenomenon by dipole–dipole interactions between two fluorophore molecules with a close distance (<10 nm). Briefly, IRC18‐LPS containing FRET acceptor (**IRC18**) and IL12/CSF1R‐MM decorated with FRET donor (Dil) were processed with membrane fusion to yield hybrid nanovesicles. Notably, the Dil emission overlapped well with **IRC18** absorbance. After the synthesized hybrid was excited under 554 nm, the Dil emission (566 nm) decreased but the **IRC18** emission (904 nm) increased, as compared to the IL12/CSF1R‐MM containing Dil and IRC18‐LPS, respectively (Figure 2D–G). These results indicate not only the FRET occurrence from Dil to **IRC18** with close molecular packing but also the successful membrane fusion.

To test the membrane labeling efficiency of **IRC18** and the targeting effect of hybrid nanovesicles, we pretreated macrophages with Dil to label the cell membrane, and the Dil‐stained membranes were coated onto IRC18‐liposome nanoparticulate substrate, forming MM (Dil)‐IRC18‐LPS. Dil/IRC18‐liposome nanovesicles (Dil/IRC18‐LPS) and Dil/IRC18 in phosphate buffered solution (Dil/IRC18‐PBS) at the same concentration acted as the controls. After 30 min co‐culture with GL261 cells, intracellular uptake of MM (Dil)‐IRC18‐LPS was assessed using confocal microscopy. The red fluorescence intensity of Dil in cells treated with MM (Dil)‐IRC18‐LPS was remarkably higher in comparison to the intensity of Dil uptake in Dil/IRC18‐LPS and Dil/IRC18‐PBS treated groups, indicating that the MM component on the outer shell of nanovesicles promoted the membrane fusion between MM (Dil)‐IRC18‐LPS nanovesicles and tumor cell membrane. (Figure 2H,I). It was worth noting that tumor cells failed to ingest the uncoated **IRC18** in PBS solution (Figure 2H lower panel); while, the liposome faciliated the uptake of Dil/IRC18‐LPS, and macrophage cell membrane further promoted uptake of MM (Dil)‐IRC18‐LPS nanovesicles (Figure 2H,I). Notably, **IRC18** and Dil co‐localized well on the GBM cell membrane for MM (Dil)‐IRC18‐LPS group and exhibited better membrane staining capability than that of Dil/IRC18‐LPS group (Figure 2H,I). These results suggested that the macrophage derived membranes promoted the uptake of nanovesicles by tumor cells, and also the design of **IRC18** as a NIR‐II emissive lipophilic cyanine is of value to achieve membrane intercalation. More importantly, MM‐derived hybrid nanovesicles contributed to the intense intercalation of **IRC18** dyes into the GBM cell membranes, which paved the way for membrane‐targeted imaging and photothermal therapy. Therefore, the **IRC18** dye is promising to be used for lipid bilayer membrane labelling in vitro and in vivo, which is of great importance for long term in vivo tracing of **IRC18** biodistribution, as well as for GBM cell membrane‐targeted photothermal therapy.

### 2.2 In Vitro PTT Effects on Tumor Cells With Nanovesicles

**Figure 3:**
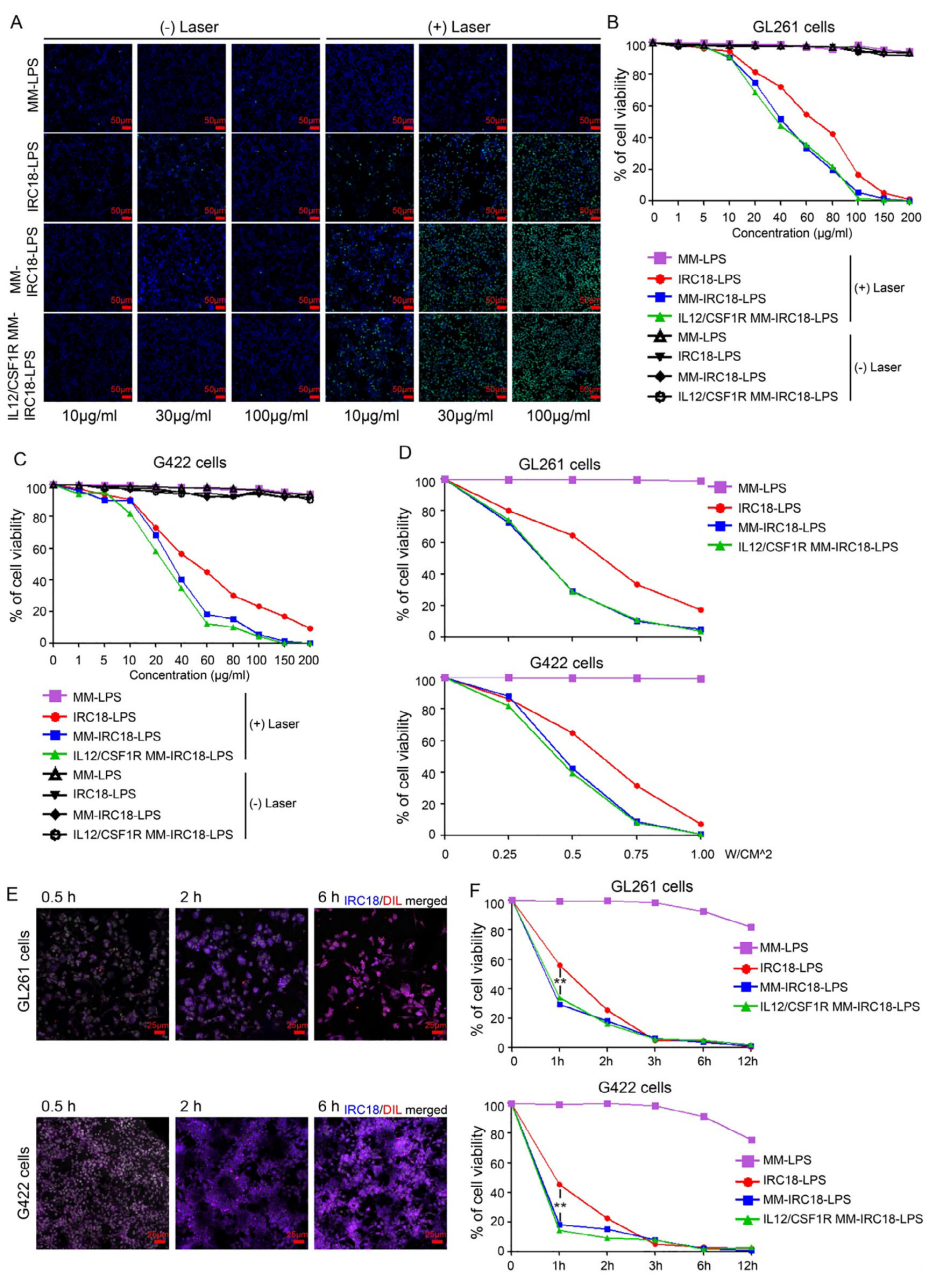
Membrane coated nanoparticles promoted PTT in vitro. A) Microscopic images showing representative DEAD dye YO‐PRO‐1 staining. GL261 cells were cultured with indicated nanoparticles for 1 h and then treated with or without laser irradiation. Green dots indicate positive staining of dead cells. B–D) Cell viability of GL261 cells and G422 cells treated with serial concentration gradients of nanoparticles (B,C) under 0.5 W cm^−2^ irradiation or with different irradiation power (D) was determined using LDH non‐radioactive cytotoxicity assay. E) Confocal microscopy imaging of GL261 and G422 cells treated with MM (DIL)‐IRC18‐LPS for indicated time duration. F) GL261 cells and G422 cells were treated with indicated nanoparticles at serial time duration and irradiated under 0.5 W cm^−2^ power for 5 min. Cell viability was determined using LDH non‐radioactive cytotoxicity assay. *p < 0.05, **p < 0.01, and ***p < 0.001 by Student's *t*‐test.

To evaluate the photothermal therapeutic effects of biomimetic nanovesicles developed in this study, we performed in vitro functional studies with different nanovesicles, including MM‐coated liposome (MM‐LPS) as control, **IRC18** integrated liposome (IRC18‐LPS), normal MM‐coated IRC18‐LPS (MM‐IRC18‐LPS), and CSF1R and GPI‐anchored IL12 overexpressed MM‐coated IRC18‐LPS (IL12/CSF1R‐MM‐IRC18‐LPS). DEAD dye YO‐PRO‐1 was applied to evaluate the in vitro NIR‐irradiation‐induced phototoxicity of samples on tumor cells. It was found that the IL12/CSF1R‐MM‐IRC18‐LPS and other formulations exhibited negligible cytotoxicity at indicated concentrations without laser irradiation, as shown by negative staining with DEAD dye (**Figure** **3**A). Under laser irradiation at 0.5 W cm^−2^ for 5 min, MM‐LPS induced no detectable cell death; while, IRC18‐LPS induced cell death in a dose‐dependent manner, as shown by the increasing nucleus staining with YO‐PRO‐1 (Figure 3A). The treatment with MM‐IRC18‐LPS and IL12/CSF1R‐MM‐IRC18‐LPS further enhanced irradiation‐induced cell death, reflecting cell membrane‐facilitated uptake of **IRC18** by tumor cells and more efficient photothermal‐induced cell death compared to IRC18‐LPS nanovesicles (Figure 3A). The cytotoxicity of these nanovesicles at different concentrations against tumor cells was evaluated using LDH non‐radioactive cytotoxicity assay. While IRC18‐LPS induced significant cell death with laser irradiation at 0.5 W cm^−2^ for 5 min, the MM‐IRC18‐LPS and IL12/CSF1R‐MM‐IRC18‐LPS groups exhibited stronger cytotoxicity with a much lower half maximal inhibitory concentration for both GL261 cells and G422 cells (Figure 3B,C). This suggests that the MMs contributed to the boosted photothermal efficacy via facilitation of cellular uptake of **IRC18** nanovesicles (Figure 2H). In addition, we treated GL261 cells and G422 cells at different laser irradiation power densities using 100 µg mL^−1^ of nanovesicles. Consistently, MM‐IRC18‐LPS and IL12/CSF1R‐MM‐IRC18‐LPS efficiently induced cell death at lower irradiation power density, as compared to IRC18‐LPS (Figure 3D). We further evaluated the incubation time for nanovesicle uptake efficiency and irradiation‐induced cytotoxicity in tumor cells. The confocal microscopy imaging revealed increasing fluorescence intensity with incubating time after incubation with MM (DIL)‐IRC18‐LPS (Figure 3E). As shown in Figure 3F, treatment of cells with MM‐IRC18‐LPS and IL12/CSF1R‐MM‐IRC18‐LPS for 1 h plus 5 min laser irradiation was enough to induce robust cell death, indicating the fast uptake of MM‐coated nanovesicles by tumor cells. These results demonstrated that the MM‐liposome hybrid nanovesicles facilitated the cellular uptake of **IRC18** and the subsequent execution of photothermal ablation of tumor cells under controllable laser irradiation. Thus, these data suggest that the MM‐liposome hybrid **IRC18** nanovesicles are promising to achieve highly efficient spatiotemporal‐controllable PTT.

### 2.3 IL12/CSF1R‐MM‐IRC18‐LPS Nanovesicles Modulate T Cell Activation and Macrophage Polarization

**Figure 4:**
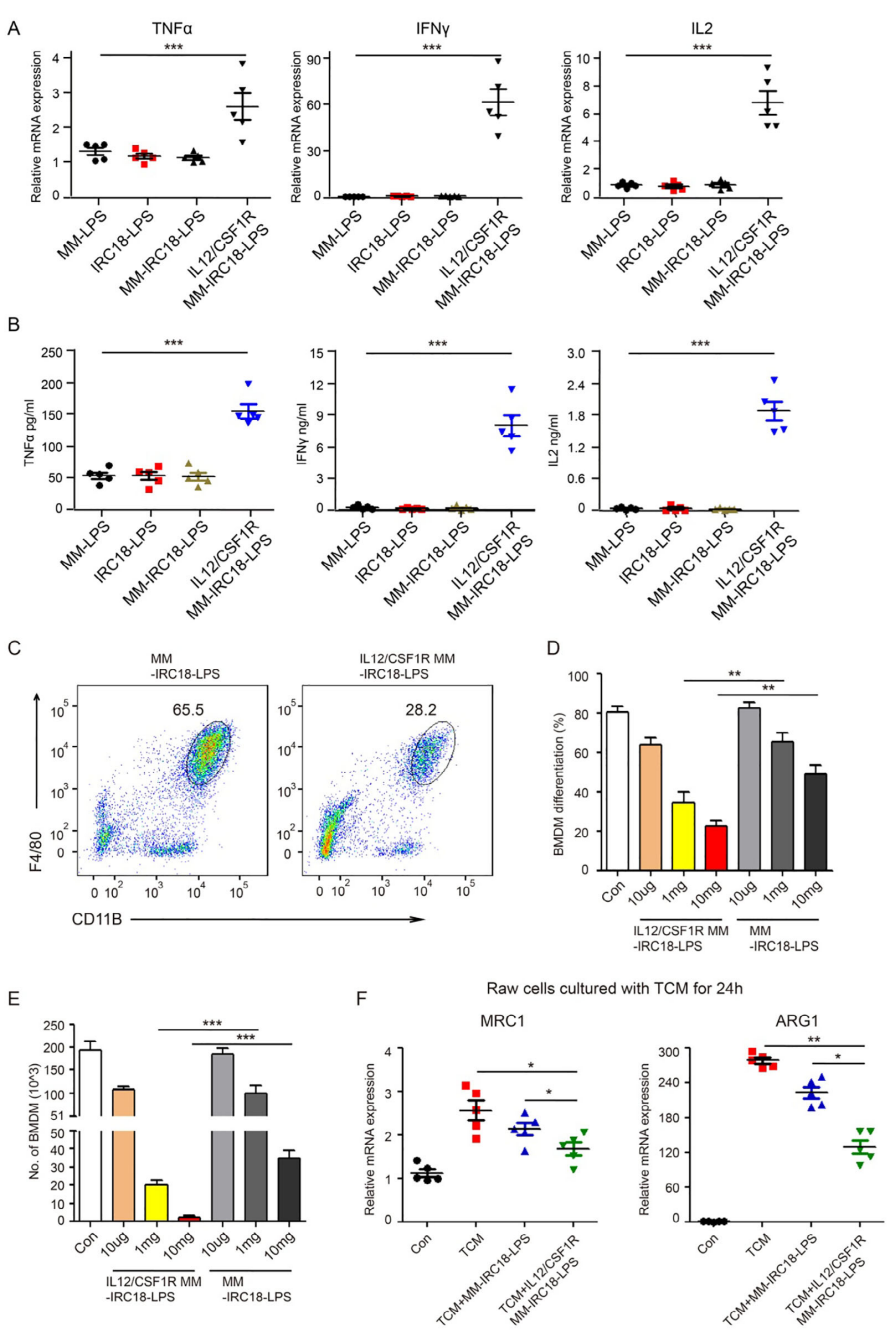
Functional characterization of CSF1R/IL12 engineered MMs. A,B) Quantitative mRNA expression (A) and protein levels (B) of TNFα, IFNγ, and IL2 in mouse spleen CD8+ T cells treated with MM‐LPS, IRC18‐LPS, MM‐IRC18‐LPS, and IL12/CSF1R‐MM‐IRC18‐LPS nanoparticles. C) Flow cytometry analysis of differentiated BMDM with CD11b and F4/80 staining. D,E) Bar graphs represent quantification of the ratio (D) and number (E) of differentiated BMDM under indicated treatments. F) Quantitative mRNA expression of the M2 macrophage markers MRC1 and ARG1 in Raw264.7 cells under indicated treatments. *n* = 5 for each group. Data are representative of three independent experiments (mean ± SEM). *p < 0.05, **p < 0.01, and ***p < 0.001 by Student's *t*‐test.

IL12 stimulates the effector functions of activated T cells by inducing cytotoxic enzymes and cytokines, mainly IFN‐γ.^[^ ^42^ ^]^ To verify whether the IL12/CSF1R‐MM‐IRC18‐LPS nanovesicles exhibit the functionality of IL12 on T cell activation, we cultured mouse spleen CD8+ T cells with MM‐LPS, IRC18‐LPS, MM‐IRC18‐LPS, and IL12/CSF1R‐MM‐IRC18‐LPS nanovesicles. In this study, the expression of effector cytokines for T cell activation was assessed by qPCR. It was found that the mRNA expression levels of TNFα, IFNγ, and IL2 were significantly upregulated in T cells treated with IL12/CSF1R‐MM‐IRC18‐LPS nanovesicles; while, these effector molecules were not significantly increased in T cells treated with MM‐LPS control, IRC18‐LPS, or MM‐IRC18‐LPS (**Figure** **4**A). These results demonstrated that the overexpressed IL12 on the cell membrane component of IL12/CSF1R‐MM‐IRC18‐LPS nanovesicles can functionally activate CD8+ T cells. In addition, the expression of these cytokines at the protein level was determined by enzyme‐linked immunosorbent assay (ELISA). Notably, consistent with mRNA expression, protein levels of TNFα, IFNγ, and IL2 in the culture supernatant were significantly increased in cells treated by IL12/CSF1R‐MM‐IRC18‐LPS nanovesicles (Figure 4B). These results indicate that the overexpressed GPI‐anchored IL12 on the IL12/CSF1R‐MM‐IRC18‐LPS nanovesicle surface maintained their biological activity well and could effectively stimulate T cell responses, which is the basis for efficient antitumor function.

Inherently expressed CSF1R on the MM has been reported to be able to bind CSF1 and act as decoy receptors to inhibit the CSF1‐CSF1R signal axis in TAM.^[^ ^40^ ^]^ In this study, we investigated whether exogenous CSF1R overexpression on MM would strengthen the inhibitory effect on CSF1‐CSF1R signal transduction. As known, CSF1 plays an important role in in vitro differentiation of bone marrow‐derived macrophages (BMDM).^[^ ^60^ ^]^ Therefore, we characterized the function of IL12/CSF1R‐MM‐IRC18‐LPS nanovesicles to inhibit BMDM differentiation in vitro. The results showed that as compared to MM‐IRC18‐LPS nanovesicles with intact MMs, IL12/CSF1R‐MM‐IRC18‐LPS nanovesicles with CSF1R‐overexpressed MMs significantly inhibited CSF1‐induced BMDM differentiation in a dose‐dependent manner (Figure 4C–E). These results indicated that CSF1R‐overexpressed MM‐coated nanovesicles can bind to CSF1; and thus, interrupt CSF1‐CSF1R signal transduction. It is known that tumor cells produce CSF1 to induce TAM differentiation and M2 polarization.^[^ ^37^ ^]^ M2 macrophages express typical markers CD206 (MRC1) and ARG1. To further evaluate the binding capability of IL12/CSF1R‐MM‐IRC18‐LPS nanovesicles to CSF1 secreted from tumor cells, we performed in vitro M2 macrophage polarization by tumor cell conditioned medium (TCM). The TCM was preincubated with MM‐IRC18‐LPS or IL12/CSF1R‐MM‐IRC18‐LPS nanovesicles and used for M2 macrophage polarization. A significant downregulation of MRC1 and ARG1 was observed, indicating the inhibition of M2 macrophage polarization by IL12/CSF1R‐MM‐IRC18‐LPS nanovesicles (Figure 4F). Taken together, these results show that the overexpressed CSF1R on the MM component of nanovesicles contributes to neutralizing CSF1 cytokine, which is of significant importance for inhibition of M2 macrophage polarization in tumors and efficient antitumor function.

### 2.4 NIR‐II Fluorescence Imaging of GBM In Vivo

**Figure 5:**
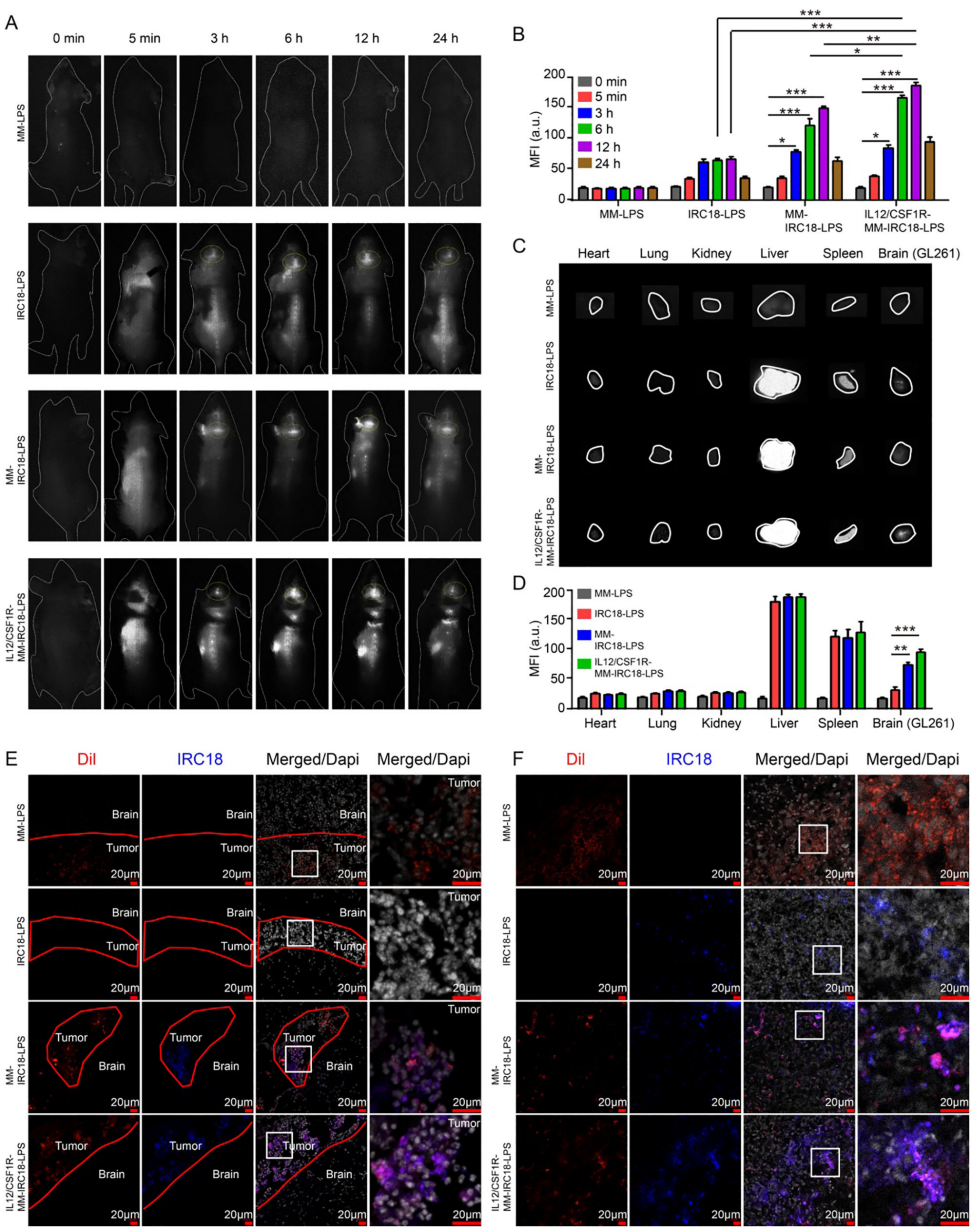
The biodistribution of MM‐coated nanovesicles. A) NIR‐II fluorescence imaging (1000LP, 100 ms) of mouse under 808 nm illumination (60 mW cm^−2^) at indicated time points post administration of IL12/CSF1R‐MM‐IRC18‐LPS, MM‐IRC18‐LPS, IRC18‐LPS, and MM‐LPS. B) Quantitative results of NIR‐II fluorescence intensity of brain tumor at different time points in (A). C) NIR‐II fluorescence imaging (1000LP, 100 ms) of dissected mouse organs under 808 nm illumination (60 mW cm^−2^). D) Quantitative results of NIR‐II fluorescence intensity of mouse organs in (C). E) Confocal microscopy imaging of mouse brains with intracranial tumors 12 h post administration of Dil‐labeled samples. Red for DIL, blue for IRC18, and grey white for cell nucleus. F) Confocal microscopy imaging of subcutaneous GL261 tumors 12 h post administration of Dil‐labeled samples. Red for DIL, blue for IRC18, and grey white for cell nucleus. *N* = 3 for each group. Data are representative of three independent experiments (mean ± SEM). *p < 0.05, **p < 0.01, and ***p < 0.001 by Student's *t*‐test.

NIR‐II fluorescence imaging has been demonstrated to be an effective modality with merits of good spatiotemporal resolution, deep penetration, and high signal‐to‐background ratio for diagnosis of GBM as well as molecular evaluation of the cytotoxic T lymphocytes in vivo.^[^ ^45^, ^47^ ^]^ In this study, we initially evaluated the BBB permeation ability of the membrane‐liposome hybrid nanovesicles using an in vitro BBB‐like bEnd.3 cell monolayer model.^[^ ^61^, ^62^ ^]^ GL261 cells were seeded at the bottom of the transwell, and the nanovesicles (MM‐LPS, IRC18‐LPS, MM‐IRC18‐LPS, and IL12/CSF1R‐MM‐IRC18‐LPS) were incubated in the upper chamber for 6 h. The fluorescence intensity of GL261 cells was measured to assess and compare the transport efficiency of these nanovesicles across the in vitro BBB. The results demonstrated that the hybridization of the macrophage membrane with IRC18‐liposome significantly enhanced transport efficiency (Figure S6A–C, Supporting Information), confirming that both MM‐IRC18‐LPS and IL12/CSF1R‐MM‐IRC18‐LPS nanovesicles successfully crossed the in vitro BBB. In this study, NIR‐II fluorescence signals of the **IRC18** were continuously recorded after intravenous injection of different formulations including MM‐LPS, IRC18‐LPS, MM‐IRC18‐LPS, or IL12/CSF1R‐MM‐IRC18‐LPS, which were used to monitor nanovescicle biodistribution and intratumor accumulation in vivo. As shown in **Figure** **5**A (left panel), there was no obvious fluorescence signal before sample injection, which suggests negligible tissue autofluorescence from mice under imaging conditions in this study. In addition, the MM‐LPS group showed negligible fluorescence signals further indicate no background interference from the source material (MM‐LPS) (Figure 5A first panel). After administration of IRC18‐containing samples, including IRC18‐LPS, MM‐IRC18‐LPS, and IL12/CSF1R‐MM‐IRC18‐LPS, respectively, NIR‐II fluorescence signals quickly appeared in the whole mouse body and gradually increased in the location of brain tumors 3h post‐injection (Figure 5A). As noted, the fluorescence brightness in brain tumor reached a plateau 12h post‐injection, which assisted in clearly pinpointing the orthotopic GBM through intact skull and scalp (Figure 5A,B). More importantly, the IL12/CSF1R‐MM‐IRC18‐LPS group demonstrated the highest brightness in brain tumors; while, the MM‐IRC18‐LPS group was brighter than the IRC18‐LPS group. Moreover, the ex vivo imaging results of major organs dissected from mice in Figure 5C,D further confirmed the in vivo imaging results (Figure 5A,B). The boosted NIR‐II fluorescence imaging in the IL12/CSF1R‐MM‐IRC18‐LPS nanovesicles group compared to both the MM‐IRC18‐LPS and IRC18‐LPS groups reflected the good performance in BBB crossing and GBM specificity with the following mechanisms, including i) the effective tumor‐homing function of MM components, ii) tumor‐targeting capability for c‐RGD peptides on PEGylated nanoplatform surface, and iii) the high level of molecular expression of CSF1 in the tumor microenvironment.^[^ ^37^ ^]^ The good accumulation of NIR‐II emissive and photothermal **IRC18** dyes in GBM tissues would contribute to enhanced performance in not only GBM diagnosis with NIR‐II fluorescence signals but also in PTT in the following studies.

To further confirm the uptake of MM‐coated nanovesicles in brain tumors, orthotopic GL261 tumor‐bearing mice were intravenously injected with MM (Dil)‐labeled nanovesicles, and their brains were sectioned and examined with fluorescence confocal microscopy. Figure 5E shows the bright emission of Dil fluorescence (red) in tumors, indicating the successful accumulation of MM (Dil)‐liposome hybrid nanovesicles. However, no obvious fluorescence appeared in the IRC18‐LPS group, suggesting negligible accumulation of IRC18‐LPS in the brain tumors (Figure 5E). Importantly, the coexistence of bright emission of Dil and **IRC18** fluorescence in the IL12/CSF1R‐MM‐IRC18‐LPS and MM‐IRC18‐LPS groups suggested that the MM components of the hybrid nanovesicles promoted the targeted accumulation of **IRC18** in the tumors (Figure 5E). To further verify the tumor targeting function of samples, we constructed subcutaneous brain tumor mouse models. As shown in Figure 5F, IRC18‐LPS samples could accumulate in tumors through enhanced penetration and retention (EPR) effects. Notably, MM‐coated nanovesicles showed much more intratumor accumulation for both IL12/CSF1R‐MM‐IRC18‐LPS and MM‐IRC18‐LPS (Figure 5F). Taken together, there is superior GBM targeting effect for IL12/CSF1R‐MM‐IRC18‐LPS to IRC18‐LPS. This indicates that the MMs together with the genetic engineering of CSF1R on the outer shell of nanovesicles contribute to efficient BBB crossing and tumor targeting. These findings were consistent with previous reports on the tumor targeted accumulation of MM‐derived nanoplatforms.^[^ ^36^, ^40^, ^44^, ^63^, ^64^ ^]^

### 2.5 Therapeutic Effects of the IL12/CSF1R‐MM‐IRC18‐LPS Nanovesicles in Orthotopic and Subcutaneous Tumor Models

**Figure 6:**
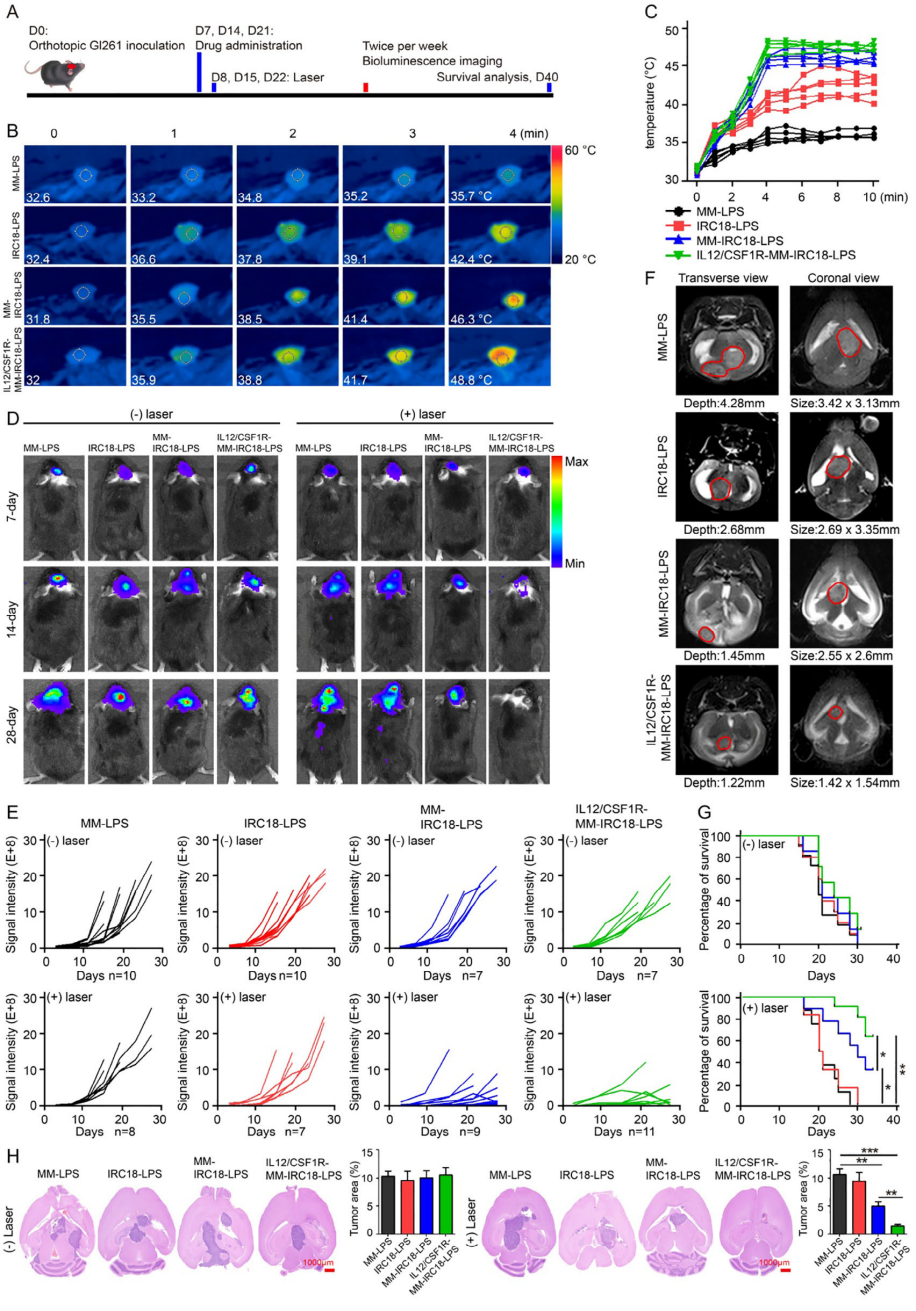
The combinatory photothermal and immuno therapy evaluation in intracranial GBM model. A) Experimental scheme for therapeutic evaluation of GBM‐bearing mice after photothermal treatment with samples including MM‐LPS, IRC18‐LPS, MM‐IRC18‐LPS, and IL12/CSF1R‐MM‐IRC18‐LPS. The mice were administrated with samples at three different time points including day 7, 14, and 21. 12 h post‐administration of samples, the mice brains were exposed to the laser irradiation (808 nm, 1.0 W cm^−2^, 10 min), for which IR thermal imaging camera was used to continuously monitor the temperature changes. Bioluminescence intensity of brain tumors was determined every 3 days using an IVIS instrument to monitor the tumor growth status. B) IR thermal images and C) the corresponding temperature curves for mice in different groups under NIR photothermal treatment. D) Representative bioluminescence images of mice bearing intracranial luciferase‐labeled GBM (GL261‐luc tumors) at days 7, 14, and 21 in different groups. E) The corresponding quantification of the total flux in luciferase signals from intracranial tumor sites as indicated in (D). F) Representative transverse and coronal view of the MRI images of mouse brain with GL261 tumor in different groups at day 28. The red circle indicates the intracranial tumor region. G) Survival curve of the GBM‐bearing mice after treatment; significance was calculated by log‐rank (Mantel–Cox) test. H) H&E histology of the representative brains of mice for each treatment. Bar graphs represent the quantitative analysis of the ratio of tumor area to the brain. Data are representative of three independent experiments (mean ± SEM). *p < 0.05, **p < 0.01, and ***p < 0.001 by Student's *t*‐test.

Encouraged by the good results on the aforementioned in vitro photothermal effects and in vivo GBM‐specific delivery of IL12/CSF1R‐MM‐IRC18‐LPS nanovesicles, we further evaluated their therapeutic effects in an orthotopic GL261 tumor model. At 7, 14, and 21 days post GL261 inoculation, mice were intravenously injected with samples (5 mg Kg^−1^ body weight), followed by photothermal treatment (laser conditions: 808 nm, 1.0 W cm^−2^, 10 min) 12 h post injection of samples, respectively (**Figure** **6**A). The intratumor hyperthermia under laser irradiation was monitored with a NIR camera, as shown in Figure 6B,C. Notably, a prompt temperature increase to ≈48 °C within 4 min of laser irradiation on the tumor site was observed for IL12/CSF1R‐MM‐IRC18‐LPS‐treated mice (Figure 6B). Under the same irradiation conditions, MM‐IRC18‐LPS‐treated mice exhibited a moderate temperature increase to ≈46 °C; while, IRC18‐LPS showed slight temperature increase to ≈42 °C (Figure 6B,C). These results demonstrated not only the good photothermal conversion capability in vivo for IRC18 dyes but also the high efficiency of BBB crossing and GBM targeting for IL12/CSF1R‐MM‐IRC18‐LPS composed of genetically engineered MMs and cRGD‐decorated PEGylated liposomes. More importantly, via spatiotemporal control of the light irradiation under NIR‐II fluorescence imaging guidance and rational administration of GBM‐specific IL12/CSF1R‐MM‐IRC18‐LPS nanovesicles, the mild hyperthermia was generated locally in GBM site, rather than in surrounding normal tissues, which offered good safety for PTT treatment.

The orthotopic GL261 tumor growth was quantitatively monitored by bioluminescence imaging, which reflects the antitumor efficacy of the nanovesicles developed in this study. In the control groups in which mice received nanovesicles without laser irradiation, the fluorescence intensity of intracranial GBM increased quickly, showing negligible therapeutic effects for nanovesicles without PTT (Figure 6D,E). In contrast, the bioluminescence intensity of GBM in the photothermal treatment groups with MM‐IRC18‐LPS and IL12/CSF1R‐MM‐IRC18‐LPS indicated attenuated tumor growth as compared to the mice without PTT treatment (Figure 6D,E). More importantly, the bioluminescence intensity of GBM for mice treated with IL12/CSF1R‐MM‐IRC18‐LPS plus PTT exhibited much slower tumor growth rate as compared to that of mice treated with MM‐IRC18‐LPS plus PTT (Figure 6D,E). Notably, at day 22, after three rounds of PTT treatment with IL12/CSF1R‐MM‐IRC18‐LPS, the tumor bioluminescence intensity even gradually decreased, suggesting dramatically suppressed tumor growth (Figure 6D,E). As magnetic resonance imaging (MRI) is an effective and non‐invasive modality to delineate the margin of GBM beneath the skull; and thus, can be used to continuously monitor treatment response,^[^ ^65^ ^]^ we further conducted MRI imaging on mice to evaluate the therapeutic efficacy in this study. The MRI results confirmed the significantly reduced 3D tumor size at day 28 in mice that received the IL12/CSF1R‐MM‐IRC18‐LPS nanovesicles plus PTT; and thus, showed superior antitumor effect to the others (Figure 6F). Consistently, mice that received IL12/CSF1R‐MM‐IRC18‐LPS plus PTT showed greatly extended survival span as compared to the other groups (Figure 6G). In addition, 4 days after the final therapy, brain samples from treated mice were collected for H&E staining. Consistent with the real‐time bioluminescent fluorescence and MRI results (Figure 6D–F), the tumor size was significantly decreased in mice with PTT (except for MM‐LPS group) as compared to mice without PTT (Figure 6H). In the photothermal therapy groups, the tumor size in mice that received MM‐IRC18‐LPS and IL12/CSF1R‐MM‐IRC18‐LPS was decreased to ≈50% and 20% of the MM‐LPS group, respectively. The better therapeutic efficacy for IL12/CSF1R‐MM‐IRC18‐LPS than MM‐IRC18‐LPS should be mainly due to the genetically overexpressed IL12/CSF1R on the MM components. Taken together, the efficient antitumor effects on GBM by the photothermal treatment with IL12/CSF1R‐MM‐IRC18‐LPS nanovesicles were due to the factors including good capacities to cross BBB and targeted accumulation in intracranial tumors, the efficient photothermal conversion to induce tumor death locally.

Apart from orthotopic GBM model, we further examined the antitumor efficacy in a subcutaneous tumor model. As shown in Figure S7A, Supporting Information, a quick increase of temperature to ≈49 °C was achieved by MM‐IRC18‐LPS and IL12/CSF1R‐MM‐IRC18‐LPS nanovesicles. Notably, the IRC18‐LPS group showed a higher temperature in subcutaneous tumors than in intracranial tumors, suggesting greater accumulation of nanovesicles in subcutaneous tumors compared to intracranial tumors. The main reason for this difference is that the blood–brain barrier hinders the accumulation of IRC18‐LPS nanovesicles in intracranial tumors; while, the EPR effect facilitates their accumulation in subcutaneous tumors. It should be noted that the in vivo photothermal results were consistent with the imaging results in Figure 5F. As to the photothermal therapeutic efficacy on subcutaneous tumors, the MM‐IRC18‐LPS and IL12/CSF1R‐MM‐IRC18‐LPS showed higher antitumor efficacy than IRC18‐LPS due to their higher photothermal heating effect (Figure S7B,D, Supporting Information). Importantly, although the nanovesicle accumulation and photothermal heating effect were similar between IL12/CSF1R‐MM‐IRC18‐LPS and MM‐IRC18‐LPS groups, the IL12/CSF1R‐MM‐IRC18‐LPS group exhibited superior antitumor efficacy compared to the MM‐IRC18‐LPS group (Figure S7B–D, Supporting Information; Figure 5F). This could possibly be due to the combined photothermal destruction of primary GBM and synergistically immunomodulatory effects of genetically overexpressed IL12/CSF1R on the macrophage membranes, which were components of the IL12/CSF1R‐MM‐IRC18‐LPS nanovesicles. Taken together, these results indicate that the IL12/CSF1R‐MM‐IRC18‐LPS nanovesicles show great potential as theranostic candidates for both GBM targeting and therapeutic applications.

### 2.6 IL12/CSF1R‐MM‐IRC18‐LPS Nanovesicles Promoted M1 Macrophage Polarization and T Cell Activation

**Figure 7:**
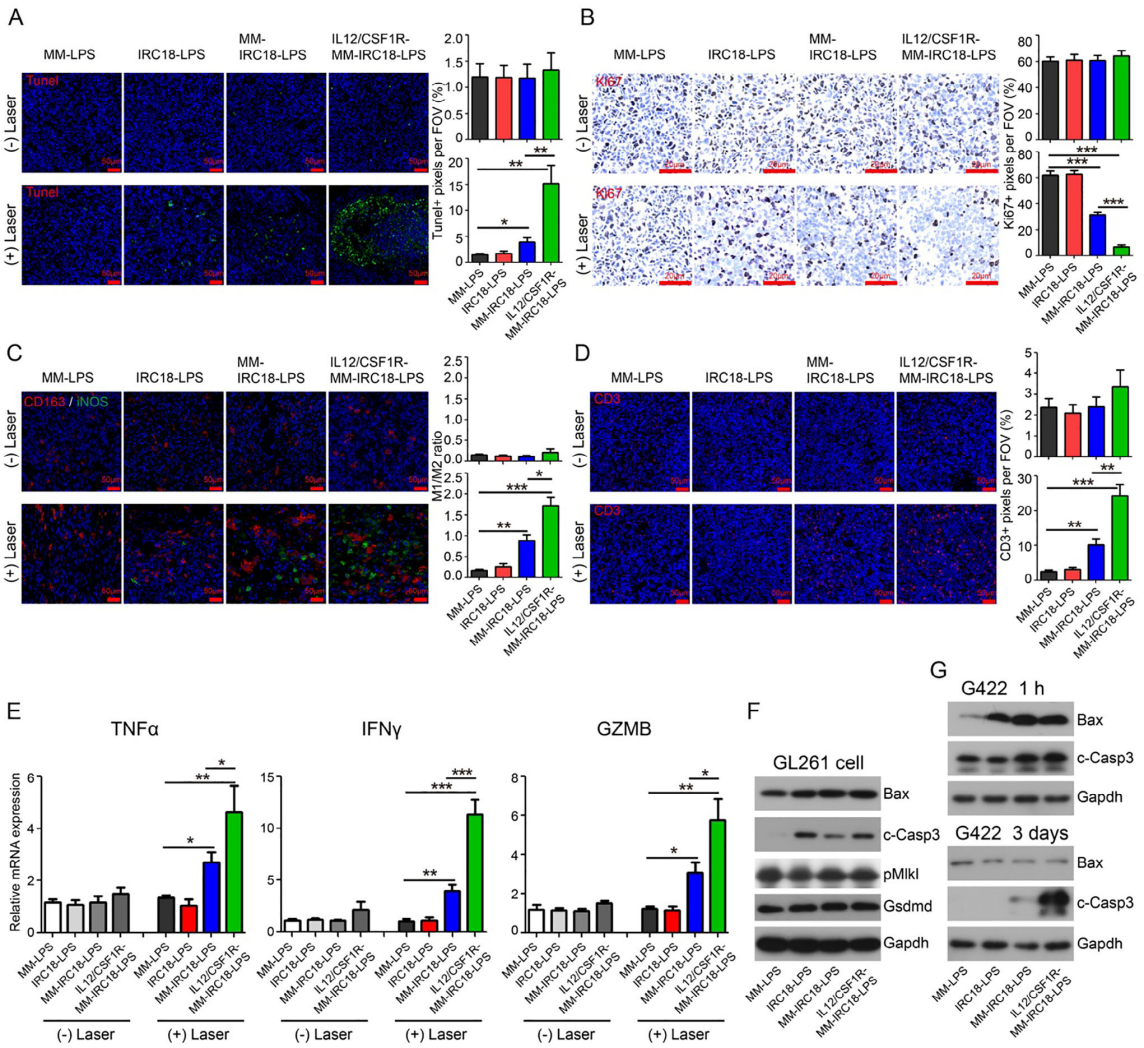
The IL12/CSF1R‐engineered membrane‐coated nanovesicles enhanced the antitumor effect through a combination of PTT and immunomodulation. A–D) Microscopic images showing the representative TUNEL staining, green dots for positive staining (A); Ki67 immunohistochemistry staining (B); M2 marker (CD163, green) and M1 marker (iNOS, red) immunofluorescence staining (C); and CD3 (red) immunofluorescence staining (D) in intracranial tumors. Blue for nucleus for all. Bar graphs represent quantitative analysis of percentages of positive staining signals per field of view (FOV). E) Quantitative mRNA expression of TNFα, IFNγ, and GZMB in tumor tissues as indicated in (A). F) Immunoblot of Bax, cleaved caspase‐3 (c‐Casp3), phosphorylated Mlkl (pMlkl), GSDMD, and Gapdh in GL261 cells that were cultured with IL12/CSF1R‐MM‐IRC18‐LPS, MM‐IRC18‐LPS, IRC18‐LPS, or MM‐LPS nanovesicles for 2 h and subjected to laser irradiation for 5 min. G) Immunoblot of Bax, c‐Casp3, and Gapdh in subcutaneous G422 tumors. Mice with subcutaneous G422 tumors were administrated with IL12/CSF1R‐MM‐IRC18‐LPS, MM‐IRC18‐LPS, IRC18‐LPS, or MM‐LPS nanovesicles, and were subjected to laser irradiation for 10 min 6 h post‐administration. Tumors were dissected 1 h post‐irradiation or 3 days post‐last irradiation. Data are representative of at least three independent experiments (mean ± SEM). *p < 0.05 and **p < 0.01 by Student's *t*‐test.

Encouraged by the aforementioned excellent anti‐GBM therapeutic efficiency of IL12/CSF1R‐MM‐IRC18‐LPS nanovesicles in vitro and in vivo, we further investigated the cellular and molecular mechanisms and aimed to verify whether the synergistic M2‐to‐M1 macrophage repolarization and enhanced T cell cytotoxicity occurred during the therapeutic processes. Consistent with the decreased tumor size, MM‐IRC18‐LPS and IL12/CSF1R‐MM‐IRC18‐LPS nanovesicles with PTT significantly induced tumor cell death, as demonstrated by strong TUNEL staining (**Figure** **7**A; Figure S7E, Supporting Information) and simultaneously inhibited tumor cell proliferation, as indicated by decreased Ki67 staining (Figure 7B; Figure S7F, Supporting Information). Importantly, compared to MM‐IRC18‐LPS plus PTT, IL12/CSF1R‐MM‐IRC18‐LPS plus PTT induced three times more cell death (TUNEL staining, 5% vs 15%) and drastically inhibited tumor cell proliferation (Ki67 staining, 30% vs 10%) (Figure 7A,B). It should be noted that nanovesicles alone without laser irradiation had negligible effects on tumor cell death and proliferation (Figure 7A,B). These results suggest three possible mechanisms: i) the intratumor photothermal effect mediated by **IRC18** was required for efficient killing of tumor cells, ii) the outer shell macrophage membranes were required for the nanovesicles to efficiently pass through the BBB and delivery of **IRC18**, and iii) CSF1R and membrane‐anchored IL12 facilitated the therapeutic effects by promoting M2‐to‐M1 repolarization and activating anti‐tumor T cell responses, thereby reprogramming the tumor immunosuppressive microenvironment and converting immunologically ‘cold’ tumors into ‘hot’ ones.

We analyzed the immunomodulation in the tumor microenvironment post treatment. The immunofluorescence staining of M1‐like (iNOS) and M2‐like (CD163) macrophages in tumor tissues revealed the significantly increased number of M1‐like macrophages and a higher ratio of M1/M2 cell numbers in intracranial GL261 tumors with IL12/CSF1R‐MM‐IRC18‐LPS plus PTT treatment than the other groups (Figure 7C). Moreover, flow cytometry analysis of the intratumoral immune cells revealed a higher ratio of M1 (CD45^+^ CD11b^hi^ CD86^+^) to M2 (CD45^+^ CD11b^hi^ CD163^+^) macrophages in GL261 tumors treated with IL12/CSF1R‐MM‐IRC18‐LPS or MM‐IRC18‐LPS plus PTT (Figure S8A,B, Supporting Information). Consistently, similar observations were discovered in the subcutaneous G422 tumor model (Figure S6G, Supporting Information). In accordance with previous in vitro findings that CSF1R on MM‐coated nanovesicles can bind to CSF1 to interrupt CSF1‐CSF1R signal transduction and inhibit M2 polarization (Figure 4C–E), our data in Figure 7C suggested the strong immunomodulatory activity of IL12/CSF1R‐MM‐IRC18‐LPS nanovesicles in combination with laser irradiation to polarize M1‐like macrophages in GBM. This meant that the TIM modulation with M2‐to‐M1 repolarization further contributed to the effective inhibition of tumor growth via immunotherapeutic mechanisms, which was in fine accordance with previous reports.^[^ ^36^, ^64^ ^]^

After studying the M2‐to‐M1 repolarization effect, we further investigated the intratumor T cells by immunofluorescence staining of CD3. MM‐IRC18‐LPS and IL12/CSF1R‐MM‐IRC18‐LPS nanovesicles plus PTT treatment significantly increased the number of intratumor CD3+ T cells in both intracranial GL261 tumors (Figure 7D) and subcutaneous G422 tumors (Figure S7H, Supporting Information), indicating the effective activation of antitumor T cell responses indeed. These results were further confirmed by flow cytometry analysis of the intratumoral CD8 (CD45^+^ CD8^+^) and CD4 (CD45^+^ CD4^+^) T cells, revealing ≈four times more CD8 and CD4 T cells in IL12/CSF1R‐MM‐IRC18‐LPS nanovesicles plus PTT‐treated tumors compared to controls (Figure S8C,D, Supporting Information). Specifically, IL12/CSF1R‐MM‐IRC18‐LPS nanovesicles plus PTT treatment substantially increased the number of intratumor T cells, indicating the good immunotherapeutic functionality of IL12 overexpressed on the outer shell of these nanovesicles (Figure 7D). As is known, TNFα, IFNγ, and GZMB are the main effector molecules of activated T cells to execute antitumor responses,^[^ ^43^ ^]^ which were determined quantitatively in this study. Consistent with an elevated number of T cells in tumors, mRNA expression levels of these genes were found to be significantly upregulated in tumor tissues after administration of MM‐IRC18‐LPS or IL12/CSF1R‐MM‐IRC18‐LPS plus PTT treatment (Figure 7E). Notably, the mRNA expression levels were more preferentially upregulated in IL12/CSF1R‐MM‐IRC18‐LPS group than in the other groups (Figure 7E). As known, IL12 could functionally activate lymphocytes, especially T cells,^[^ ^42^ ^]^ which was corroborated in this study as well. Mechanistically, the IL12 anchored on the outer shell of nanovesicles activated the T lymphocytes and initiated a cascade of phenotypic alterations, typified by increased production of pro‐inflammatory cytokines or effector molecules including TNFα, IFNγ, and GZMB.^[^ ^66^ ^]^ Our data in Figure 7; Figure S6, Supporting Information showed that IL12/CSF1R‐MM‐IRC18‐LPS nanoplatforms drastically reshaped the TIM, favoring cytotoxic T cells in combination with M2‐to‐M1 repolarization. Simply put, these results revealed that the CSF1R and IL12 overexpressed membrane‐coated photothermal sensitive **IRC18** nanovesicles succeeded in integrating PTT, M2‐to‐M1 macrophage repolarization, and effector T cell activation for combinatorial membrane‐targeted PTT and immunotherapy of GBM in an all‐in‐one nanoplatform.

To further investigate the molecular mechanisms underlying tumor cell death induced by PTT, the core regulators of different types of cell death were analyzed. Under the in vitro laser irradiation, we found that Bax and activated caspase‐3 were upregulated; while, pMLKL and GSDMD remained unchanged (Figure 7F), indicating that cell apoptosis accounted for PTT‐induced cell death; while, cell necroptosis or pyroptosis was not involved in this condition. In addition, in the in vivo subcutaneous tumors, Bax and activated caspase‐3 were upregulated 1 h after laser irradiation by MM‐IRC18‐LPS and IL12/CSF1R‐MM‐IRC18‐LPS (Figure 7G). However, in subcutaneous tumors 3 days after the last treatment, only activated caspase‐3 was detected with IL12/CSF1R‐MM‐IRC18‐LPS treatment (Figure 7G). These results indicate that cancer cell membrane‐targeted PTT induced immediate apoptotic cell death through the hyperthermia effect on tumor cells, and genetically engineered MMs on the outer shell of nanovesicles exerted long‐term effects on tumor cells possibly through immunomodulation.

Although IL12/CSF1R‐MM‐IRC18‐LPS nanovesicles induced significant M2‐to‐M1 macrophage repolarization and effector T cell activation in tumors, these nanovesicles did not induce significant immune changes in peripheral blood, as assessed by flow cytometry analysis for blood B cells, CD8^+^ T cells, CD4^+^ T cells, regulatory T cells (Treg), and macrophages (Figure S8E,F, Supporting Information). Notably, our results showed raised IFNγ production and serum aspartate transaminase (AST) levels at day 3 after administration of IL12/CSF1R‐MM‐IRC18‐LPS nanovesicles (Figure S9A,B, Supporting Information). However, the levels of IFNγ and AST were significantly lower than those in mice administrated with IL‐12 (0.1 µg per mice), a tolerable dose for mice (Figure S9A,B, Supporting Information).^[^ ^48^ ^]^ These results underscored the ability of IL12/CSF1R‐MM‐IRC18‐LPS to enhance targeted anti‐tumor function; while, avoiding off‐target toxicity. We also compared body weight changes and observed that mice treated with MM‐LPS or IRC18‐LPS started to lose weight 15 days after tumor inoculation, possibly due to the tumor growth and resultant brain damage. However, the body weight of mice treated with IL12/CSF1R‐MM‐IRC18‐LPS nanovesicles remained mostly unchanged, with a tendency to gain weight by the end of the experiment (Figure S9C, Supporting Information). These data suggested that IL12/CSF1R‐MM‐IRC18‐LPS nanovesicles not only contributed to efficient anti‐tumor responses but also had acceptable toxicity. In addition, a systematic analysis of hematological parameters was conducted to evaluate the physiological status of the mice which were treated with IL12/CSF1R‐MM‐IRC18‐LPS nanovesicles. The blood urea nitrogen (BUN), albumin (ALB), blood total protein (TP), creatinine (Crea), hemoglobin (HGB), red blood cell (RBC), white blood cell (WBC), and lymphocyte percentage (LY%) showed negligible fluctuations during the treatment period (Figure S9D, Supporting Information), which suggested that the nanovesicles exhibit good biosafety. H&E staining of the main organs (heart, liver, lungs, kidneys, and brain) showed no notable pathological abnormalities, implying the high histocompatibility of these nanovesicles (Figure S9E, Supporting Information). Further, to monitor the excretion of these nanovesicles in vivo, feces were collected after drug administration.^[^ ^67^ ^]^ NIR‐II fluorescence imaging showed active excretion of these nanovesicles within the first 4 days, with no detectable signal by day 6 post‐administration (Figure S10A, Supporting Information). These findings demonstrated that the nanovesicles could be excreted via the liver/gallbladder system, which was corroborated by their significant accumulation in the liver (Figure 5C). Further, ex vivo imaging of the major organs dissected from the mice confirmed the clearance of the drugs by day 7.5 after administration (Figure S10B, Supporting Information). Taken together, these data suggest that IL12/CSF1R‐MM‐IRC18‐LPS nanovesicles exhibited excellent biosafety and biocompatibility without inducing systemic inflammation.

## 3 Conclusion

To the best of our knowledge, we first developed an efficient glioblastoma treatment paradigm of membrane‐targeted mild photothermal‐immunotherapy with lipophilic NIR‐II emissive probe (**IRC18**)‐loaded liposome and genetically engineered IL12/CSF1R‐macrophage membrane hybrid nanovesicles. **IRC18** has a similar lipophilic and octadecyl structure to the commercial membrane probe Dil and extended conjugation length with NIR‐I photothermal and NIR‐II emissive capabilities, which can intercalate glioblastoma cell membrane through lipid membrane fusion. The biomimetic IL12/CSF1R‐macrophage membrane and c‐RGD‐decorated and PEGylated liposome synergistically achieved efficient blood–brain‐barrier crossing and tumor targeting, with efficient enrichment of nanovesicles in glioblastoma. This further contributed to clear pinpointing of tumor margin and even molecular visualization of tumoral microenvironment via non‐invasive NIR‐II fluorescence imaging through intact skull and scalp. Notably, the good membrane‐intercalating capability of **IRC18** boosted photothermal therapy in tumors, which succeeded in achieving mild hyperthermia for high therapeutic efficacy and minimized side effects. More importantly, the targeted enrichment of CSF1R and IL12 in glioblastoma blocked the CSF1‐CSF1R signaling axis to promote M2‐to‐M1 repolarization and activated the antitumor T cell responses, respectively, which expedited the execution of glioblastoma. Collectively, this NIR‐II fluorescence imaging‐guided membrane‐targeted mild photothermal and multiple‐immunomodulatory “all‐in‐one” nanoplatform represents an efficient theranostic paradigm for glioblastoma treatment.

While our study demonstrates the promising potential of IL12/CSF1R‐MM‐IRC18‐LPS nanovesicles for NIR‐II fluorescence imaging‐guided membrane‐targeted mild photothermal‐immunotherapy of glioblastoma, several limitations should be acknowledged. First, the current study is primarily based on cell and mouse models, and further validation in larger animal models is necessary to confirm the safety and efficacy of this approach in humans. Second, although the nanovesicles showed efficient BBB penetration and tumor targeting, the heterogeneity of glioblastoma and individual variations in BBB integrity may affect the consistency of drug delivery in clinical settings. Third, the long‐term biosafety and potential immunogenicity of the genetically engineered macrophage membranes need to be thoroughly evaluated in future studies. Fourth, the efficiency of IL12/CSF1R overexpression in macrophages, as well as the yield and purity of isolated cell membranes, should be optimized as these are critical factors influencing the functionality of the nanovesicles.

## Conflict of Interest

The authors declare no conflict of interest.

## Supporting information



Supporting Information

## Data Availability

The data that support the findings of this study are available in the supplementary material of this article.
