# Supplementary material for: Genetically Engineered IL12/CSF1R‐Macrophage Membrane‐Liposome Hybrid Nanovesicles for NIR‐II Fluorescence Imaging‐Guided and Membrane‐Targeted Mild Photothermal‐Immunotherapy of Glioblastoma
Source: Adv Sci (Weinh). 2025 Apr 25;12(23):2500131. doi: 10.1002/advs.202500131 (PMC12199386; doi:10.1002/advs.202500131)
Supplement: Supplementary file 1 — Supporting Information [file ADVS-12-2500131-s001.docx]

Supporting Information

Genetically Engineered IL12/CSF1R-Macrophage Membrane-Liposome Hybrid Nanovesicles for NIR-II Fluorescence Imaging-Guided and Membrane-Targeted Mild Photothermal-Immunotherapy of Glioblastoma

Pengfei Chen^1,#,^*, Yue Liu^2,#^, Haiyan Huang^2^, Menglong Li^2^, Hui Xie^3^, Chen Yu^2^, Shubham Roy^2^, Jingsi Gu^4^, Jian Jin^4^, Jing Cheng^4^, Yinghe Zhang^2^, Kai Deng^1^, Lixin Du^5^, Bing Guo^2,^*

Experimental Section

Reagents

1,2-Dipalmitoyl-sn-glycero-3-phosphocholine (DPPC), 1-palmitoyl-2-hydroxy-sn-glycero-3-phosphocholine (MPPC), 1,2 distearoyl-snglycero-3-phosphoethanolamine-N-Methoxy polyethyleneglycol-2000 (DSPE-PEG_2000_) were purchased from Avanti Polar Lipids Inc. 1,2-distearoyl-sn-glycero-3-phosphoethanolamine-N-[maleimide(polyethylene glycol)-2000]5-thiolated cyclo(Arg-Gly-Asp-D-Phe-Lys(mpa)) peptide (c-RGD) (DSPE-PEG_2000_-cRGD) was purchased from [Xi'an ruixi Biological](https://www.bing.com/ck/a?!&&p=a63ca2c07b8b7913JmltdHM9MTcwMTczNDQwMCZpZ3VpZD0yYjdiMjRhNy04N2ZjLTY4ZGItMWFhNy0zNGRmODZiZjY5Y2EmaW5zaWQ9NTI4NQ&ptn=3&ver=2&hsh=3&fclid=2b7b24a7-87fc-68db-1aa7-34df86bf69ca&psq=%e8%a5%bf%e5%ae%89%e7%91%9e%e7%a6%a7%e7%94%9f%e7%89%a9%e7%a7%91%e6%8a%80%e6%9c%89%e9%99%90%e5%85%ac%e5%8f%b8%e8%8b%b1%e6%96%87&u=a1aHR0cDovL3d3dy54YXJ4YmlvLmNvbS9wcm8vcHJvZHVjdC5odG1s&ntb=1) (China). DMEM (G4511) was purchased from Wuhan Servicebio Technology. Fetal bovine serum (FBS, A5670701), penicillin and streptomycin (15140148), anti-CD3ε (16-0031-86,) and anti-CD28 (16-0281-86) were purchased from ThermoFisher. Recombinant mouse M-CSF (416-ML) was purchased from R&D Systems. RIPA lysis buffer (P0013C) and YO-PRO-1 (C2022) were obtained from Beyotime Biotechnology. Trizol reagent (15596026CN) was from InvitrogenTM. PrimeScript™ RT Master Mix (RR036A), TB Green® Premix Ex Taq™ (Tli RNaseH Plus) (RR420) were from Takara Bio. Protease inhibitor cocktail (P8340) was purchased from MERCK. CD8a microbead kit (130-126-707) was from Miltenyi Biotec. Mouse IL-12 p70 ELISA Kit (PK00018) was purchased from Abclonal Technology. Rapid gold BCA protein assay kit (A55862) was from ThermoFisher. CytoTox 96 non-radioactive cytotoxicity assay kit (G1780) was from Promega. TUNEL assay kit (G1504) was purchased from Wuhan Servicebio Technology. Anti-CD3 (ab16669), anti-CD163 (ab182422), anti-iNOS (ab210823), and anti-Ki67 (ab15580) were purchased from Abcam. Fluorescence labeled Anti-mouse F4/80 (12-4801-82) was purchased from Invitrogen^TM^. Anti-mouse CD45 (147706), Anti-mouse CD11b (101212), Anti-mouse CD86 (105018), Anti-mouse CD163 (156708), Anti-mouse B220 (103222), Anti-mouse CD4 (100529), Anti-mouse CD8 (162306), Anti-mouse CD25 (113711), and Anti-mouse Foxp3 (118904) were purchased from Biolegend.

Synthesis and Characterization

The NMR and ESI-MS spectra are measured by a Bruker AV-400 spectrometer and LTQ Orbit rap XL instruments, respectively. Density functional theory (DFT) and time-dependent DFT (TDDFT) calculations were conducted by the Gaussian 09 program. DLS test and ζ-potential were conducted on Malvern Nanoziser (Nano ZS90, Malvern, UK) at ambient temperature. The morphology and size of nanoparticles was studied by field emission transmission electron microscopy (TEM, JEM-1400, JEOL, Japan), and Zetasizer (Nano ZS90, Malvern, UK) DLS instrument, respectively. Confocal laser scanning microscopy (CLSM) images were obtained using a STELLARIS 8 confocal microscope (Leica, Germany). The 808 nm NIR laser was purchased from Changchun Lashi Optoelectronic Technology Co., Ltd. The UV-Vis and Fluorescence spectra were monitored with 1 cm quartz cuvette through Shimadzu (Suzhou, China) UV-1800 spectrophotometer and F-7000 spectrophotometer (HATACHI, Shizuoka, Japan), respectively.

The synthetic route for compound **IRC18** was as follows:

**Scheme S1.** Synthetic route for compound **IRC18**. (i) iodooctadecane, acetonitrile, 83 °C; (ii) 2-chloro-3-(hydroxymethylene)-cyclohex-1-enecarboxaldehyde, sodium acetate, acetic anhydride, 60 °C.

*Synthesis of* ***compound 2****.* Weighing 2, 3, 3-trimethylindole (795 mg, 5 mmol), iodooctadecane (3.80 g, 10 mmol) was dissolved in 10 mL of acetonitrile and the mixture was refluxed with stirring at 83 ℃ for 15 h. Then the reaction system was cooled to room temperature and filtered, and the solid was washed with ethyl acetate to give a pink solid **compound 2** (1.94 g, 3.6 mmol, 72%). ^1^H NMR (400 MHz, DMSO) δ 7.99 – 7.93 (m, 1H), 7.86 – 7.81 (m, 1H), 7.66 – 7.59 (m, 2H), 4.43 (t, *J* = 7.6 Hz, 2H), 2.83 (s, 3H), 1.87 – 1.76 (m, 2H), 1.53 (s, 6H), 1.40 (s, 2H), 1.23 (s, 28H), 0.85 (t, *J* = 6.7 Hz, 3H). ^13^C NMR (101 MHz, CDCl_3_) δ 195.53, 141.69, 141.04, 130.18, 129.57, 123.37, 115.33, 77.40, 77.08, 76.76, 54.68, 50.32, 33.61, 31.96, 30.55, 29.73, 29.70, 29.66, 29.59, 29.50, 29.46, 29.40, 29.18, 28.59, 27.99, 26.88, 23.25, 23.04, 22.73, 17.06, 14.17, 7.47. HRMS(ESI^+^) m/z calcd for C_29_H_50_N^+^ (M+H)^+^ 412.39378, found 412.39362.

*Synthesis of* ***IRC18****.* (269.2 mg, 0.5 mmol)1, (43 mg, 0.25 mmol) 2-chloro-1-formyl-3-hydroxymethylene cyclohexene condensate and sodium acetate (21.3 mg, 0.26 mmol) were dissolved in acetic anhydride and the mixture was heated under nitrogen at 60 ℃ for 3 h. At the end of the reaction, the mixture was cooled down to room temperature and filtered. The solids were washed with sodium bicarbonate buffer, then rinsed with water and finally dried under vacuum to obtain green powder **IRC18** (81.5 mg, 0.075 mmol, 30% yield). ^1^H NMR (400 MHz, DMSO) δ 8.25 (d, *J* = 14.1 Hz, 2H), 7.63 (d, *J* = 7.3 Hz, 2H), 7.48 – 7.40 (m, 4H), 7.29 (t, *J* = 7.1 Hz, 2H), 6.32 (d, *J* = 14.3 Hz, 2H), 4.22 (t, *J* = 6.4 Hz, 4H), 2.69 (t, *J* = 6.8 Hz, 4H), 1.85 (dd, *J* = 12.7, 7.1 Hz, 2H), 1.77 – 1.68 (m, 4H), 1.66 (s, 12H), 1.36 – 1.20 (m, 61H), 0.82 (t, *J* = 6.8 Hz, 6H). ^13^C NMR (101 MHz, DMSO) δ 170.42, 164.71, 141.59, 135.99, 134.11, 133.11, 120.42, 118.53, 117.04, 114.00, 102.82, 92.85, 41.14, 40.14, 39.93, 39.71, 39.50, 39.29, 39.07, 38.86, 35.65, 27.24, 26.91, 19.58, 18.83, 18.67, 18.30, 18.11, 17.86, 17.62, 17.20, 16.95, 12.62. HRMS(ESI^+^) m/z calcd for C_66_H_104_ClN_2_^+^ (M+H)^+^ 959.78826, found 959.78833.


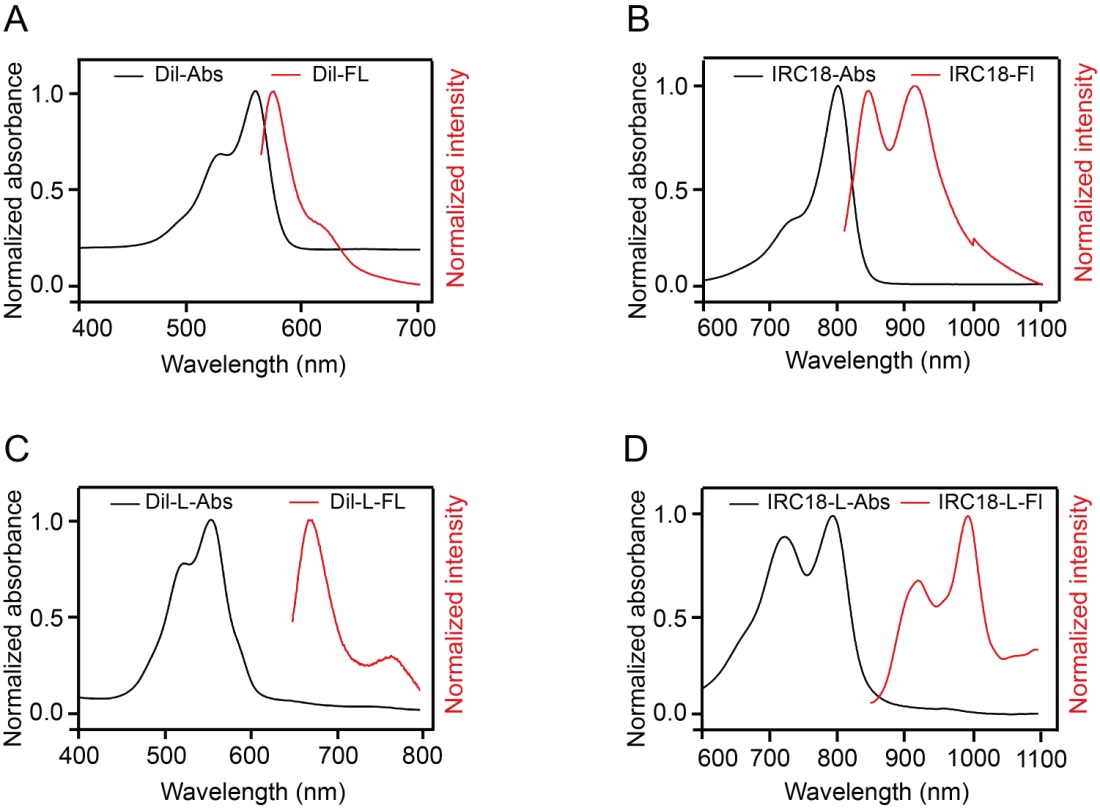


**Figure S1.** Density functional theory calculations of dyes. normalised UV absorption and emission of Dil **(A)** and IRC18 **(B)** dyes in DMSO solution; normalised UV absorption and emission of liposomes made of Dil **(C)** and IRC18 **(D)** dyes in PBS solution.

Cell culture and genetic engineering

GL261, G422 and Raw264.7 cells were maintained with DMEM containing 10% (vol/vol) FBS, penicillin (100 U/ml) and streptomycin (100 μg/ml) at 37 °C in a 5% CO2 incubator.

For genetic engineering, prepare the lenti virus expressing CSF1R and GPI-anchored IL12 with virus titer of 1×10^8^ PFU/ml. 2×10^5^ Raw264.7 cells were plated in 12-well plate overnight, change the medium with 250μl fresh medium and 250μl virus plus 1μg/ml polybrene, change the medium 12 hours later and culture the cells for 2 days. The cells were then subjected to puromycin and G418 selection for 7 days.

Isolation of macrophage membrane

Isolation of macrophage membrane was performed as previously described.[[1](#_ENREF_1)] 1 × 10^8^ CSF1R/IL12 engineered or control Raw264.7 cells were harvested and washed with 1× PBS three times and then re-suspended in 5 ml cold hypotonic lysis buffer (20 mM Tris-HCl, pH 7.4; 10 mM MgCl2; 10 mM KCl; protease inhibitor cocktail). The cells were then subjected to ultrasonication (power 200W, work 5 seconds and stop 5 seconds for 10 minutes) in ice-bath. The cells were centrifuged at 3,200g for 5 min at 4°C to collect the supernatant. The pellet was subjected to ultrasonication again and the supernatant was collected after centrigugation. The supernatant collections were pooled together and centrifuged at 20,000g, 4°C, 30 min and discard the pellet. The supernatant was then ultracentrifuged at 80,000g, 4°C, 2 hours (LE-80K, Beckman Coulter, USA).The pellet was resuspended in 500 μl cold PBS and subjected to ultrasonication (42 kHz, 100W, 4 min). The concentrition of membrane proteins was determined with rapid gold BCA protein assay kit .

Synthesis of IL12/CSF1R-MM-IRC18-LPS

Liposomes were synthesized by thin film hydration method.[[2](#_ENREF_2)] Firstly, the mass ratio of 16:2:2:1:1 DPPC, MPPC, IRC18, DSPE-PEG2000, DSPE-PEG2000-cRGD was put into the test tube, and the raw material was dissolved with trichloromethane, and then sealed with a sealing film for 5 min, and then placed in a 65 ℃ water bath to be rapidly heated and shaken, and then touched to the whole test tube was slightly hot, and then put into vortex mixer, turned on the nitrogen, and blew nitrogen into the test tube to form a film. Then put the test tube into the vortex mixer, and blow nitrogen into the test tube to form a film. After the film is formed, seal it with a sealing film, and then blow the next tube and repeat the above operation. After all the blown film, poke 3 small holes with scissors and put it into a vacuum desiccator to evacuate the chloroform for 3-4 h. Remove the test tube from the desiccator, add 1 mL of Phosphate Buffered Saline (PBS), blow it evenly, and ultrasonic vibrate it for 10 min. A small extruder was then used to extrude the polycarbonate film back and forth through the 400 nm and 200 nm 10 times. Finally, the liposomes were dialyzed through a dialysis bag to remove the unencapsulated dye. IRC18 liposome (IRC18-LPS) was finally obtained.

To coat the macrophage membranes with IRC18-LPS by a direct extrusion method, 1 mg IRC18-LPS was mixed with 1 mg genetically engineered macrophage membranes by vortexing and sonication (30% amplitude, 30-second pulse on/off, 2 min) to 1 ml, freeze-thaw cycling three times, and then extruded through 400 and 200 nm polycarbonate membranes 10 times, respectively, to facilitate the fusion process. After extrusion, IL12/CSF1R-MM-IRC18-LPS was subjected to an ultrafiltration unit in order to remove free components with a molecular weight cutoff (MWCO) of 3500 (i.e., 3500 kDa).


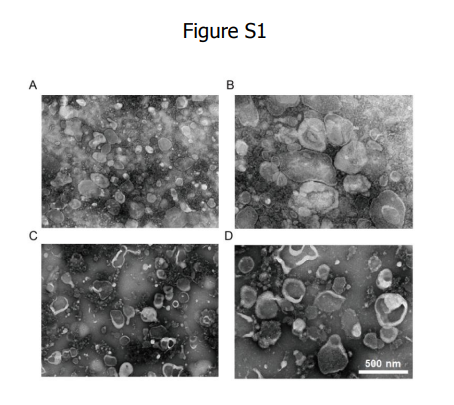


**Figure S2.** TEM images of macrophage membranes isolated by the four methods (**A-D**), scale bar 500 nm. (**A**) power 200W, work 3 seconds and stop 3 seconds for 5 minutes. (**B**) power 200W, work 3 seconds and stop 3 seconds for 10 minutes. (**C**) power 200W, work 5 seconds and stop 5 seconds for 5 minutes. (**D**) power 200W, work 5 seconds and stop 5 seconds for 10 minutes.

Elucidation of the Hybridization Mechanism.

Hybridization of the liposome and macrophage membrane was verified by a fluorescence resonance energy transfer (FRET) study.[[2](#_ENREF_2), [3](#_ENREF_3)] Dil, a fluorescence donor, was incubated with macrophage membrane at 37°C for 30 minutes to obtain Dil-labeled macrophage membranes. Before freezing and thawing, the fluorescence signals of Dil-labeled membraness and IRC18-labeled liposomes were separately detected. After three freeze-thaw cycles, the changes in fluorescence of Dil and ICR18 before and after freezing were observed to compare the fluorescence signals in the Dil and NIR-C_12_ channels and validate the successful fusion of liposomes with endogenous drug carriers. The fluorescence changes of Dil and IRC18 before and after freezing were observed using a fluorescence spectrometer.

Fluorescence stability of nanoparticles

To study the photostability, the nanoparticles were pre-incubated with different mediums including water, PBS buffer, and 20% FBS solution. Subsequently, the samples were subjected to continuous irradiation by 808 nm laser at a power intensity of approximately 100 mW/cm^2^. After these treatments, fluorescence images at 0 min and 24 hours were recorded using a NIR-II imaging system.

Quantum yield measurement

NIR-II quantum yield characteristics of nanoparticles were determined.[[4](#_ENREF_4)] The NIR-II quantum yield (QY) was determined using a previously reported method.[[5](#_ENREF_5)] The emission spectra were measured using a fluorescence spectrometer. To calculate the detailed QY values, a typical IR-II dye, IR-1061, with a NIR-II QY of 0.75% was chosen as the reference standard. For the working solutions, IR-1061 dye and the test samples were separately diluted with dichloromethane (DCM) and PBS, resulting in 808 nm absorbance densities of approximately 0.06, 0.07, 0.08, 0.09, and 0.10. Subsequently, the detailed absorption and fluorescence spectra of these samples were measured, and the emission integration of peak area in the range of 900-1500 nm was obtained based on the data, along with the 808 nm absorbance. A similar method was used to further characterize the NIR-II QY of these nanoparticles in aqueous solutions. To calculate the NIR-II QY, the slope of the linear relationship between the emission integration of peak area and absorbance intensity was determined. The detailed NIR-II QY values were then obtained using the following formula：

$$\Phi_{sample}=\Phi_{ref}*\frac{{Slope}_{sample}}{{Slope}_{ref}}*{(\frac{n_{sample}}{n_{ref}})}^{2}$$

where Slope_sample_ and Slope_ref_ are the slopes between the emission peak area and absorbance of the sample to be tested, the standard IR-1061, and *n_sample_* and n_ref_ represents the refractive index of the corresponding solvents (mainly including DCM and PBS).


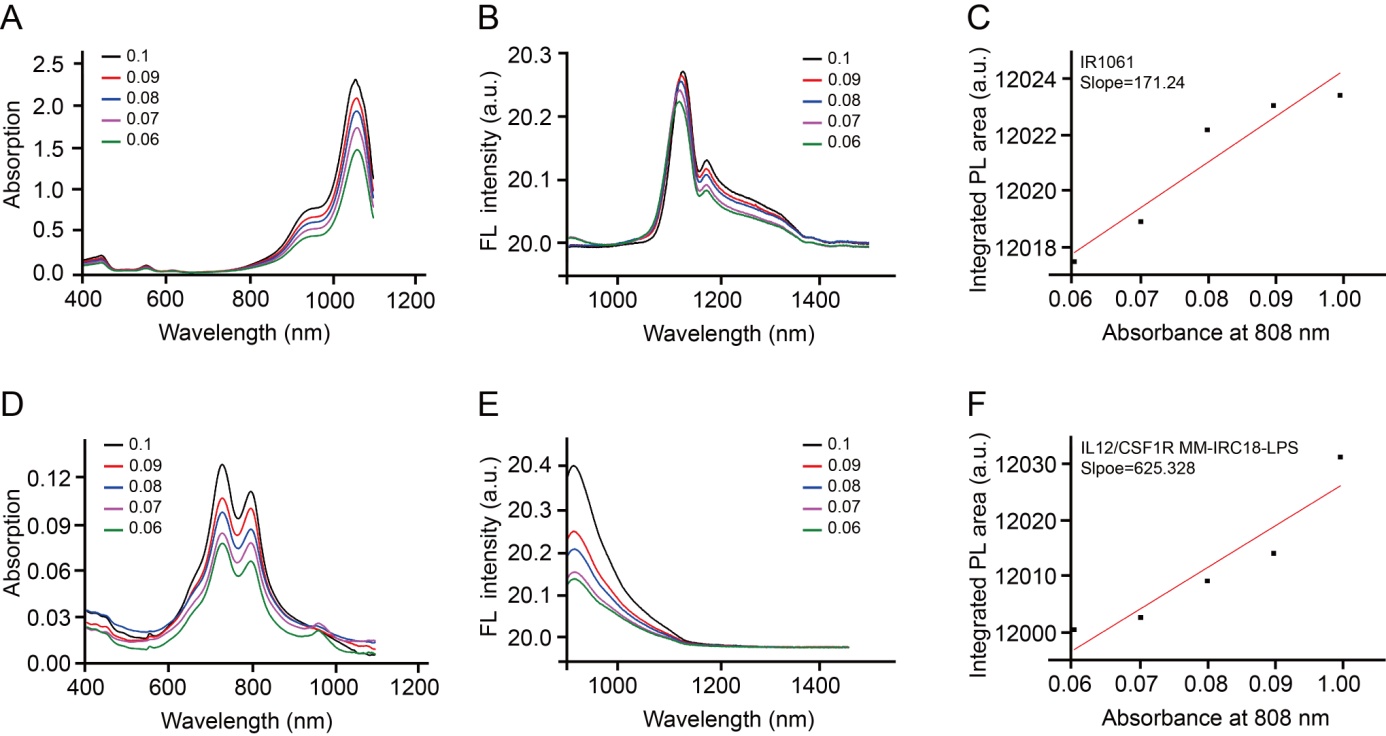


**Figure S3.** Fluorescence quantum yield of IL12/CSF1R-MM-IRC18-LPS. UV-visible-near infrared spectra of IR1061 **(A)**, IL12/CSF1R-MM-IRC18-LPS **(D)**; emission spectra of IR1061 **(B)**, IL12/CSF1R-MM-IRC18-LPS **(E)**; integrated IR1061 **(C)**, integrated emission area of IL12/CSF1R-MM-IRC18-LPS **(F)** as a function of absorbance at 808 nm.

Photothermal Conversion Efficiency Calculation

The photothermal conversion efficiency (PCE, η) for nanoparticles in PBS were measured according to the reported works.[[6](#_ENREF_6)] First, nanoparticles was irradiated by 808 nm laser with power of 1 W/cm^2^ for 15 min. Then, these samples were cooling down to room temperature with the temperature recorded by an infrared camera. The value of ηwas calculated through the following equation:

$\eta=\frac{hA\left( T_{max}-T_{surr} \right)-Q_{Dis}}{I(1-{10}^{-A}`)}$ (1)

where *h* and *A* indicate the heat transfer coefficient and surface area of the container, respectively. *T_Max_* and *T_Surr_* represent the maximum steady-state and room temperature, *I* represents the laser power (1 W/cm^2^), *A* is absorbance of the specimen at 808 nm, *Q_Dis_* is the heat dissipation from the laser mediated by the solvent and the container, which could be calculated by Equation (2), the value of *hA* could be calculated through the Equation (3).

$Q_{Dis}=\frac{mc(T_{Max(water)}-T_{surr})}{\tau_{s(water)}}$ (2)

$\tau_{s}=\frac{mc}{hA}$ (3)

where m is mass of the aqueous solution (1.0 g) and c is the heat capacity of water (4.2 J/g), τs is the time constant which could be calculated by Equations (4) and (5):

$t={-\tau}_{s}ln(\frac{T_{RT}-T_{Surr}}{T_{Max}-T_{Surr}})$ (4)

$\theta=\frac{T_{RT}-T_{Surr}}{T_{Max}-T_{Surr}}$ (5)

*T_RT_* is the real temperature in this cooling process, *θ* is the driving force temperature.

Plasmids construction

The coding sequence of mouse CSF1R and GPI-anchored IL12 were synthesized and cloned into vector pLVX-EF1a-IRES-Puro and pLVX-EF1a-IRES-Neo, respectively.

Evaluation of the cellular uptake of nanoparticles

GL261 or G422 cells were seeded at a density of 5 ×10^5^ cells per well in 6-well plates overnight. The cells were then incubated with Dil-labeled nanoparticles: MM(Dil)-IRC18-LPS, Dil/IRC18-LPS, or Dil/IRC18 solution in PBS for 1 hour or different time duration as needed. After incubation, the cells were rinsed twice with PBS, fixed with 4% formaldehyde for 5 min, and analyzed under a confocal fluorescent microscope.

Dead Cell staining

GL261 cells were seeded at a density of 2 ×10^5^ cells per well in 24-well plates overnight. The cells were then incubated with nanoparticles IL12/CSF1R-MM-IRC18-LPS, MM-IRC18-LPS , IRC18-LPS, or MM-LPS at indicated concentrations. At the end of treatment, the cells were washed three times with PBS to clear free nanoparticles, add 250μl culture medium without phenol red. The cells were irradiated for 5 min with an NIR laser (808 nm, 0.5 Wcm^-2^). After irradiation, add 1μM DEAD dye YO-PRO-1 to each well and keep the plate in CO2 incubator for 30 min. Images were obtained with fluorescence microscope.

Cell viability assay

The cell cytoxicity was measured as LDH release using a CytoTox 96 non-radioactive cytotoxicity assay kit (Promega) according to the manufacturer’s instructions. GL261 or G422 cells were seeded at a density of 2 ×10^5^ cells per well in 24-well plates overnight. The cells were then incubated with nanoparticles CSF1R/IL12-MM-IRC18-LPS, MM-IRC18-LPS , IRC18-LPS, or MM-LPS at indicated concentrations or different time duration as needed. At the end of treatment, the cells were washed three times with PBS to clear free nanoparticles, add 250μl culture medium without phenol red. The cells were irradiated for 5 min with an NIR laser (808 nm, 0.5 Wcm^-2^) and kept in CO2 incubator for 1 hour. Transfer 50μl aliquots from all test to a 96-well flat bottom plate, add 50μl of the CytoTox 96® Reagent to each sample, incubate in dark for 30 minutes at room temperature, add 50μl of Stop Solution to each well, and record the absorbance at 490nm. Percent cell viability was caculated as (1-Experimental LDH release/Maximum LDH release) ×100.

Animal studies

Female C57BL/6 and Kunming mice were purchased from Guangdong Medical Laboratory Animal Center. All mice were maintained in specific pathogen-free conditions. All animal experiments were performed in compliance with the guide for the care and use of laboratory animals and were approved by the institutional biomedical research ethics committee of Guangdong Medical University (GDY2202329).

Subcutaneous tumor cell graft

Kunming mice at the age of 6-8 weeks were used for subcutaneous tumor cell graft. 1 × 10^6^ G422-luciferase cells (G422-luc) were suspended in 100 μl PBS, and injected subcutaneously into right flank of Kunming mice. The growth of G422-luc tumors were monitored twice a week by bioluminescence imaging (IVIS imaging system, PerkinElmer, USA). When the tumor volume reached about 100 mm^3^, mice were divided randomly into 4 groups (6 mice per group): MM-LPS, IRC18-LPS, MM-IRC18-LPS, CSF1R/IL12-MM-IRC18-LPS. The mice were intravenously injected with 100 μL of the respective formulations at day 7, 14, and 21 post tumor cell graft. The hair around the tumor site was carefully removed with hair removal cream, and the tumors were irradiated for 10 min with an NIR laser (808 nm, 0.5 Wcm^-2^) 6 hours after each administration. The tumor growth was monitored through bioluminescence intensity every three days using an IVIS instrument.

Orthotopic intracranial tumor cell graft

1×10^6^ GL261-luc cells suspended in 2μl PBS were microinjected into the right striatum of C57BL/6 mouse brain (0.5 mm anterior and 2 mm lateral from the bregma, and 2 mm deep from the skull surface) as previously described. At day 7 the mice with successful intracranial tumor development (as assessed by *in vivo* bioluminescence) were divided randomly into 8 groups (6 mice per group): MM-LPS, IRC18-LPS, MM-IRC18-LPS, CSF1R/IL12-MM- IRC18-LPS with or without laser irradiation. The mice were intravenously injected with 100 μL of the respective formulations at day 7, 14, and 21 post tumor cell graft. The hair around the tumor site was carefully removed with hair removal cream, and the tumors were irradiated for 10 min with an NIR laser (808 nm, 1.0 Wcm^-2^) 12 hours after each administration. The tumor growth was monitored through bioluminescence intensity every three days using an IVIS instrument.

In vivo biodistribution of CSF1R/IL12-MM-IRC18-LPS nanoparticles

The mice with successful intracranial tumor development (as assessed by *in vivo* bioluminescence) were intravenously injected with various nanoparticles (5 mg/kg, 100 μL). Noninvasive NIR-II fluorescence imaging (1000LP, 100 ms) of mouse under 808 nm illumination (60 mW cm^−2^) at 0 min (before administration), 5 min, 30 min, 3 hours, 6 hours, 12 hours, and 24 hours post administration was performed. After 24 hours post administration, the mice were sacrificed, and the major organs were dissected for NIR-II fluorescence imaging.

Isolation of CD8+ lymphocytes and in vitro stimulation

Mouse spleen was mechanically disrupted and single cells were passed through a 40 μm filter. CD8+ lymphocytes were isolated with CD8a T cells enrichment kit (Miltenyi Biotec) according to manufacturer’s instructions. 24-well tissue culture plates were coated with anti-CD3ε (10 μg/ml) and anti-CD28 (10 μg/ml) for 12 hours, and excess antibodies were aspirated before T cell stimulation. CD8+ lymphocytes were stimulated with 100μg/ml MM-LPS, IRC18-LPS, MM-IRC18-LPS, IL12/CSF1R-MM-IRC18-LPS nanoparticles for 72 hours.

***In vitro* BBB-crossing test**

To construct an *in vitro* BBB model, 2 x 10^5^ bEnd.3 cells were seeded in the upper well of 6-well transwell plates and cultured for 5 days. A TEER value exceeding 200 Ω·cm^2^ was considered indicative of successful BBB formation. Subsequently, GL261 cells were seeded in the lower chamber. Nanovesicles were added to the upper chamber and incubated for 6 hours. The fluorescence intensity of GL261 cells in the lower chamber was analyzed using flow cytometry.

Enzyme-linked immunosorbent assay

The membrane was isolated from the genetic engineered Raw264.7 cells and the membrane protein concentration was determined with rapid gold BCA protein assay kit. For quantification of membrane-anchored IL12 in isolated membrane component, IL12 concentration in 1-10μg of isolated membrane was determined with mouse IL12 ELISA Kit according to the manufacturer’s instructions.

Hematoxylin-Eosin (H&E), immunofluorescence and immunohistochemistry staining

Mouse brains were dissected at the end of experiment and paraffin embedded. 6 μm-thick brain slices were deparaffinized, rehydrated, endogenous peroxidase blocked, antigen-retrieved, blocked with 5% BSA.

For immnofluorescence assay, block the samples with 3% bovine serum albumin at room temperature for 30 min. The sections were incubated overnight at 4°C with primary antibodies anti-CD3 (1:500), anti-CD163 (1:200), anti-iNOS (1:200). The slides were washed in PBS for 3 times and incubated with corresponding fluorescence-labeled secondary antibodies.

For immunohistochemistry staining, block the samples with 3% bovine serum albumin at room temperature for 30 min. The sections were incubated overnight at 4°C with primary antibodies against Ki67 (1:500). The slides were washed in PBS for 3 times and covered with HRP-labeled secondary antibody of the corresponding species of the primary antibodies for 1 hour at room temperature. After 3 washes, the slides were stained with freshly prepared DAB color developing solution. The slides were washed with distilled water and stained with hematoxylin solution for nuclei. Dehydrate and transparent the samples by alcohol, n-butanol and xylene. Seal the slides and the results were interpreted under a white light microscope.

Staining was quantified using at least 5 randomly selected 20× fields of view. All stainings were quantified using NIH ImageJ analysis software with the same threshold for each set (http://rsb.info.nih.gov/nih-image/).

BMDM differentiation

Tibia and femur bones were extracted from 6-8 weeks old mice and bone marrow was flushed using PBS. Bone marrow cells were cultured in DMEM supplemented with 10% FBS, 1% penicillin and streptomycin and 5ng/ml M-CSF plus different concentrations of MM-IRC18-LPS or IL12/CSF1R-MM-IRC18-LPS nanoparticles for 4 days. The differentiated BMDM at day 4 were typsin digested, followed by centrifugation at 200g for 5 min and washed in PBS. The cells were blocked in 2% BSA for 10 min and stained with fluorescence-labeled anti-mouse CD11b (1:100) and anti-mouse F4/80 (1:100) for 30 min. The cells were washed twice with PBS, resuspended in PBS containing 1% BSA and detected with BD FACS Aria II ﬂow cytometer (Franklin Lakes, NJ, USA).

TUNEL assay

Cell apoptosis in tumor tissues was performed with TUNEL assay Kit (Wuhan Servicebio Technology) according to the manufacturer’s instructions. Images were obtained with confocol fluorescence microscope.

Western blots analysis

Tumor tissues or cells were homogenized in RIPA lysis buffer and lysed on ice for 30 min. Supernatants were collected after centrifugation (12000g) at 4 °C for 30 min. Lysates or isolated membranes were separated by sodium dodecyl sulfate-polyacrylamide gel electrophoresis (SDS-PAGE) and blotted onto a polyvinylidene fluoride membrane. After blocking, the membrane was probed with the indicated primary antibodies at 4 °C overnight. Rinse the membrane with PBST 5 min for 3 times, and incubate with horseradish peroxidase conjugated secondary antibody at room temperature for 1 hour in 5% nonfat-dried milk, followed by visualization using an enhanced chemiluminescence (ECL) detection system. Band intensity was analyzed by Image J software. Relative band intensity of interest was normalized to the corresponding intensity of GAPDH.

RNA extraction and qPCR analysis

Tissues or cells were homogenized and preserved in Trizol reagent and total RNA was extracted by trichloromethane extraction, isopropanol precipitation, ethanol rinsing and Rnase-free water dissolution. Total RNA was reverse transcribed into complementary DNA (cDNA) and analyzed with SYBR Premix Ex Taq (Takara) on 7500 Real-Time PCR System (Applied Biosystems). Relative mRNA expression level for each gene was assessed by normalization to the expression of the housekeeping gene Rpl13a.

Primer list:

CSF1R-F: TGTCATCGAGCCTAGTGGC

CSF1R-R: CGGGAGATTCAGGGTCCAAG

IL12A-F: CAATCACGCTACCTCCTCTTTT

IL12A-R: CAGCAGTGCAGGAATAATGTTTC

IL12B-F: TGGTTTGCCATCGTTTTGCTG

IL12B-R: ACAGGTGAGGTTCACTGTTTCT

MRC1-F: CTCTGTTCAGCTATTGGACGC

MRC1-R: TGGCACTCCCAAACATAATTTGA

ARG1-F: CTCCAAGCCAAAGTCCTTAGAG

ARG1-R: AGGAGCTGTCATTAGGGACATC

mTNFα-F:CAGGCGGTGCCTATGTCTC

mTNFα-R:CGATCACCCCGAAGTTCAGTAG

IFNγ-F: ATGAACGCTACACACTGCATC

IFNγ-R: CCATCCTTTTGCCAGTTCCTC

IL2-F: TGAGCAGGATGGAGAATTACAGG

IL2-R: GTCCAAGTTCATCTTCTAGGCAC

GZMB-F: CCACTCTCGACCCTACATGG

GZMB-R: GGCCCCCAAAGTGACATTTATT

Rpl13a-F: GGGCAGGTTCTGGTATTGGAT

Rpl13a-R: GGCTCGGAAATGGTAGGGG

**Flow cytometry:**

Tumors were carefully dissected and chopped into small pieces and disintegrated with 0.01% (w/v) Liberase TH and 100 U/ml DNase I in RPMI 1640 at 37℃ for 30 min. The tissue pieces were spun up and down several times to facilitate dissection. Cells were then filtered through a 40-μm cell strainer and washed with 5 ml wash buffer (1 X PBS with 2 mM EDTA and 0.5% BSA), followed by centrifugation at 200g for 5 minutes. Cells were resuspended with 5 ml ACK lysis buffer, holding on ice for 5 minutes. The cells were then washed with 10 ml wash buffer twice. To isolate peripheral blood monocytes, 20 μl of tail vein blood was mixed with 20 μl of 3 mg/ml EDTA solution. Then, 0.5 ml of ACK lysis buffer was added, and the mixture was incubated on ice for 5 minutes. The cells were washed with 2 ml of wash buffer, and the flocculent was carefully discarded.

The cells were blocked with CD16/32 antibody for 10 minutes and stained with fluorescent-conjugated antibodies. Antibodies to CD45, CD11b, CD86, and CD163 were used for intratumoral mcarophage staining. Antibodies to CD45, CD4, and CD8a were used for intratumoral lymphocytes staining. Antibodies to CD45, B220, CD4, and CD8a were used for blood lymphocytes staining. Antibodies to CD25, Foxp3, CD4, and CD8a were used for blood Treg staining. Foxp3/Transcription Factor Staining Buffer Set (ThermoFisher, 00-5523-00) was used for intracellular staining. Antibodies to CD45, CD11b, and F4/80 were used for blood macrophage staining. The cells were washed, resuspended in PBS containing 1% BSA, and analyzed by BD FACS Aria II flow cytometer (Franklin Lakes, NJ, USA).

Statistical Analysis

GraphPad Software (the 5th version) was used to perform statistical analysis and graph development. Data are representative of three independent experiments, with at least three samples per experiment (mean ± SEM). A two-tailed Student’s *t* test was used to evalue the significance. Survival curves were presented using Kaplan-Meier method and significance was calculated by log-rank (Mantel-Cox) test. p values < 0.05 were considered statistically significant. *p < 0.05, **p < 0.01, ***p < 0.001.


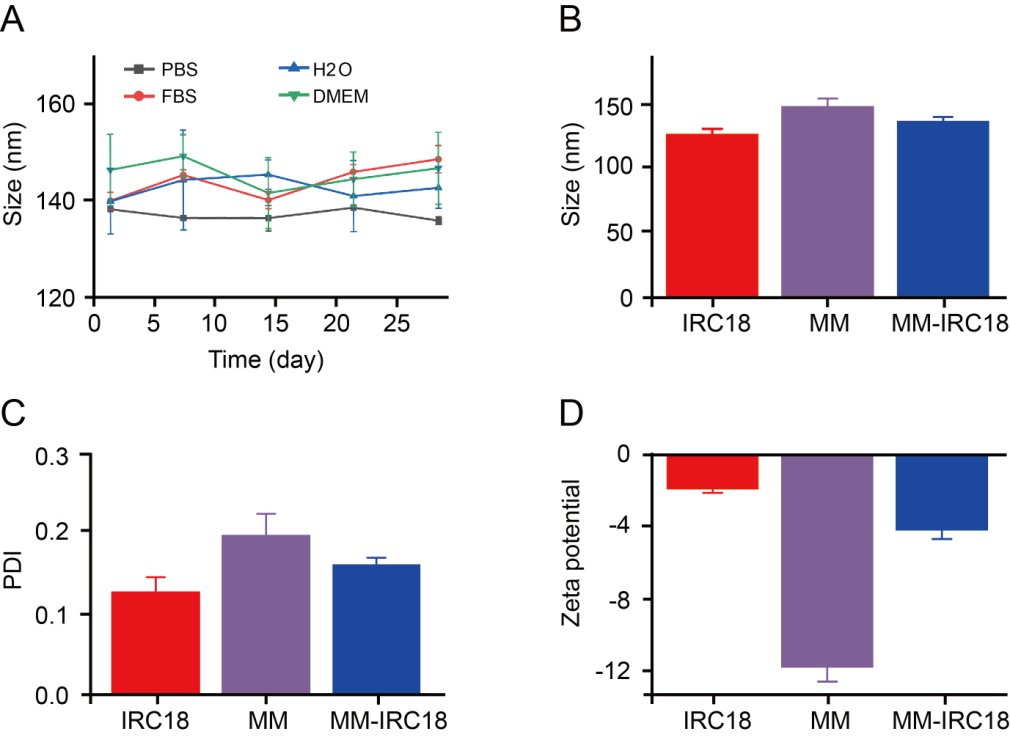


**Figure S4.** **(A)** Variation in particle size of IL12/CSF1R-MM-IRC18-LPS over a four-week period in different media; IRC18-LPS, IL12/CSF1R-MM (abbreviated MM), IL12/CSF1R-MM-IRC18-LPS (abbreviated MM-IRC18-LPS) for **(B)** particle size, **(C)** PDI, **(D)** Zeta potential.


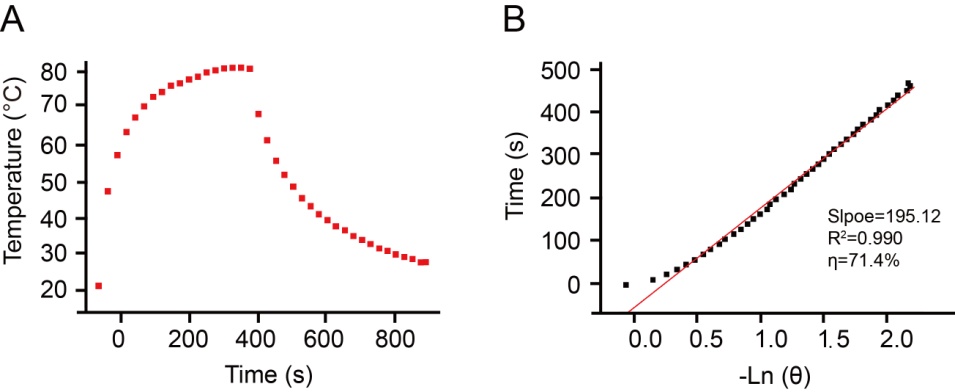


**Figure S5.** **(A)** Temperature profile of 50 μg/mL IL12/CSF1R-MM-IRC18-LPS in PBS; **(B)** Plot of the logarithm of the cooling time versus the driving force temperature of IL12/CSF1R-MM-IRC18-LPS during the photothermal conversion process.


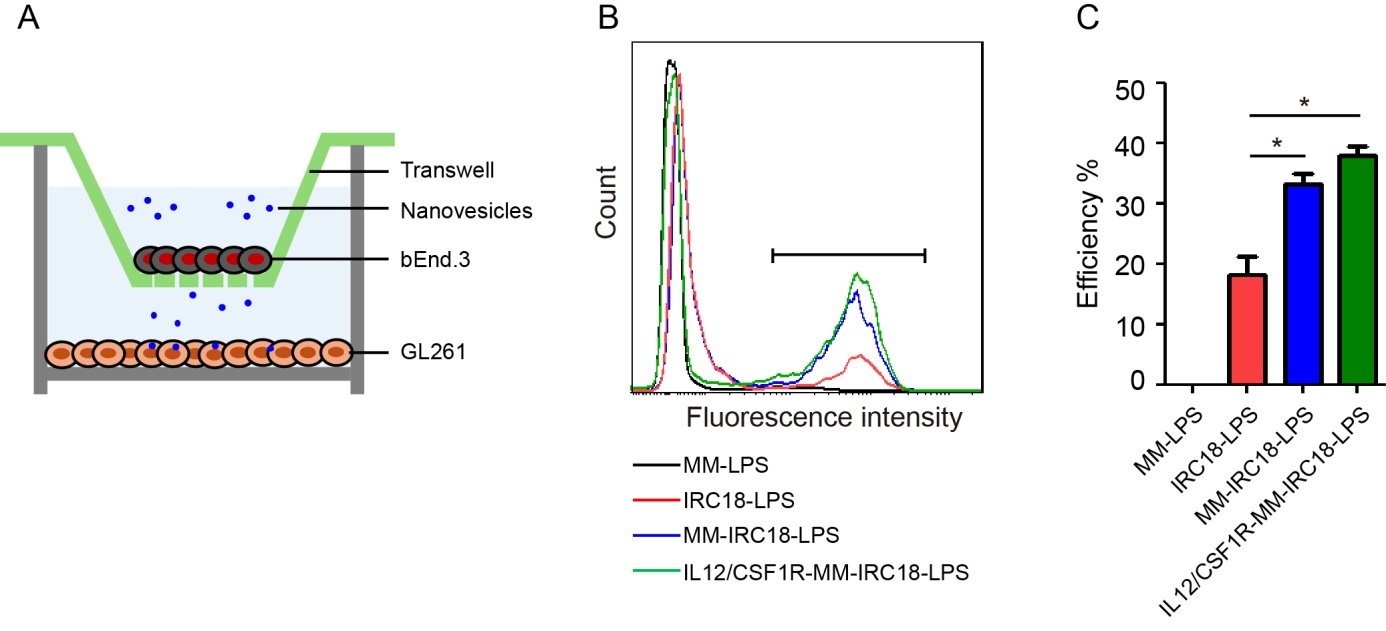


**Figure S6.** **(A)** Schematic diagram of the *in vitro* BBB model. **(B)** Flow cytometric analysis showing the uptake of nanoparticles by GL261 cells in the bottom well after incubation with MM-LPS, IRC18-LPS, MM-IRC18-LPS, and IL12/CSF1R-MM-IRC18-LPS in the upper chamber for 6 hours. **(C)** Statistic analysis of the percentages of GL261 cells that were positive for IRC18. Data are representative of three independent experiments (mean ± SEM ). *p < 0.05 by Student’s t test.


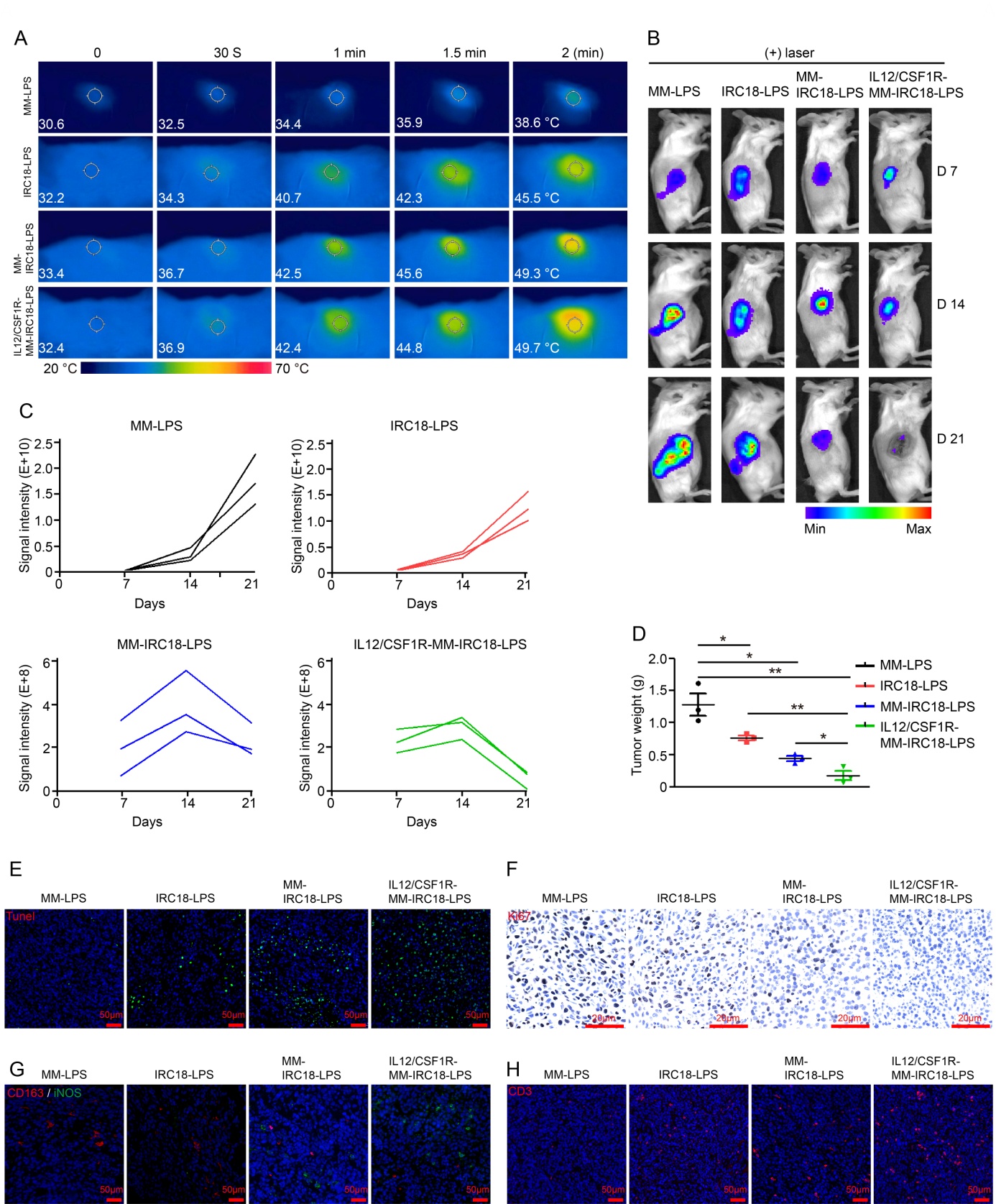


**Figure S7.** The therapeutic effects of macrophage membrane coated nanoparticles in mouse subcutaneous tumor model. (**A**) Infrared thermal images of tumors under NIR laser irradiation. (**B**) Representative bioluminescence images of subcutaneous G422-luc tumors at day 7, 14, and 21 for each teatment. (**C**) Corresponding quantification of the total flux in luciferase signals from subcutaneous G422-luc tumors as indicated in (B), n=3. (**D**) Quantification of the tumor weight of subcutaneous G422-luc tumors as indicated in (B) , n=3. (**E-H**) Microscopic images showing the representative TUNEL staining, green dots for positive staining (E), Ki67 immunohistochemistry staining (F), M2 marker (CD163, green) and M1 marker (iNOS, red) immunofluorescence staining (G), CD3 (red) immunofluorescence staining (H) in subcutaneous G422-luc tumors as indicated in (B). Blue for nucleus for all. Data are representative of three independent experiments (mean ± SEM ). *p < 0.05, **p < 0.01 by Student’s t test.


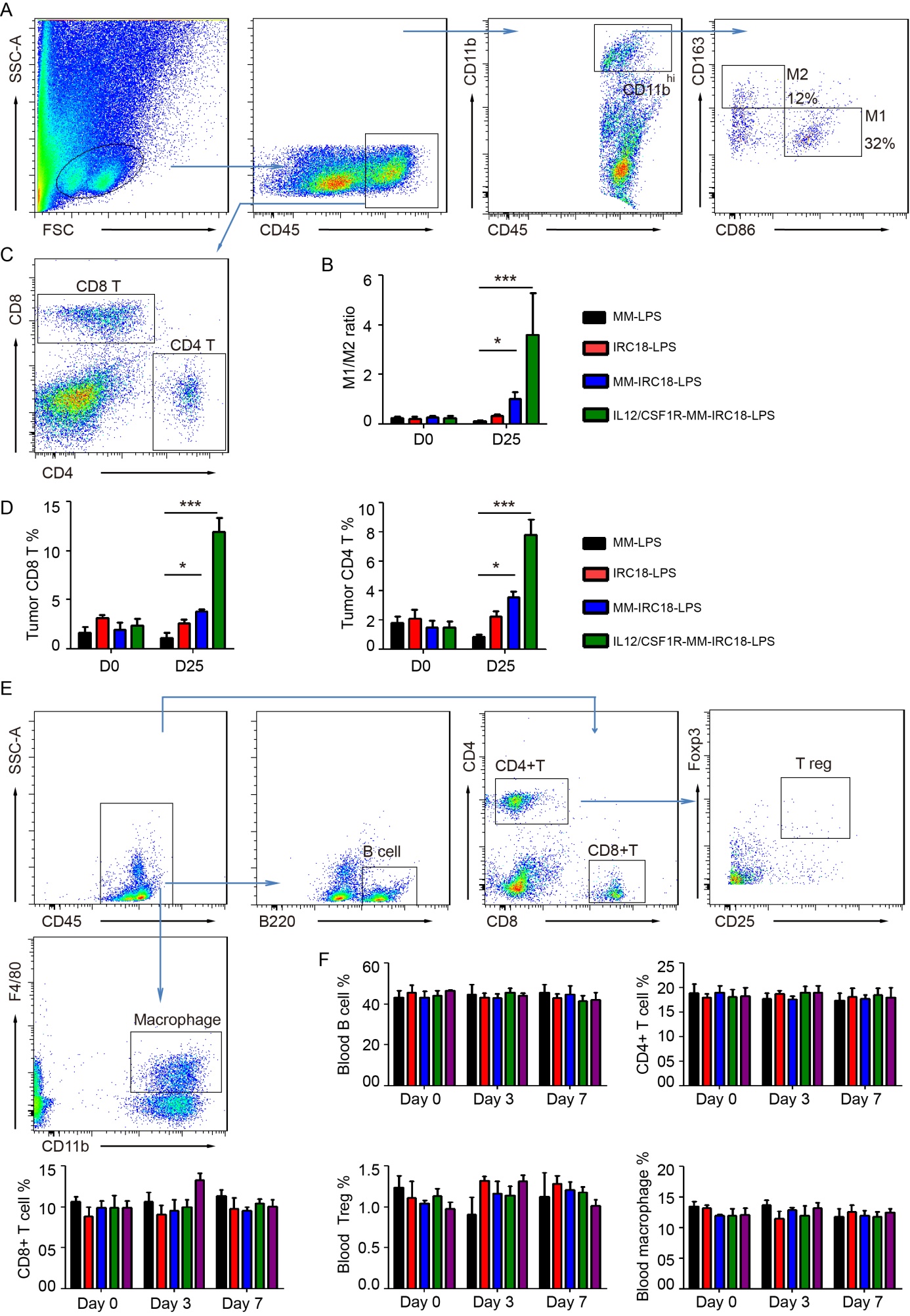


**Figure S8.** The characteristics of immune cells in tumors and blood. (**A and C**) Flow cytometry analysis of intratumoral M1 macrophage (CD45^+^ CD11b^hi^ CD86^+^), M2 macrophage (CD45^+^ CD11b^hi^ CD163^+^), CD4 T cells (CD45^+^ CD4^+^), and CD8 T cells (CD45^+^ CD8^+^). (**B**) The ratio of M1 macrophage percentage to M2 macrophage percentage in tumor tissues, as determined by flow cytometry analysis in (A). **(D)** Statistic analysis of the percentages of CD4 T cells and CD8 T cells in tumor tissues, as determined by flow cytometry analysis in (C). (**E**) Flow cytometry analysis of peripheral blood mononuclear cells, B cells (CD45^+^ B220^+^), CD4 T cells (CD45^+^ CD4^+^), CD8 T cells (CD45^+^ CD8^+^), regulatory T cells (Tregs, CD45^+^ CD4^+^ CD25^+^ Foxp3^+^), and macrophage (CD45^+^ CD11b^+^ F4/80^+^). **(F)** Statistic analysis of the percentages of B cells, CD4 T cells, CD8 T cells, Tregs, and macrophages in mouse blood, as determined by flow cytometry analysis in (E). Data are representative of three independent experiments (mean ± SEM). *p < 0.05, ***p < 0.001 by Student’s t test.


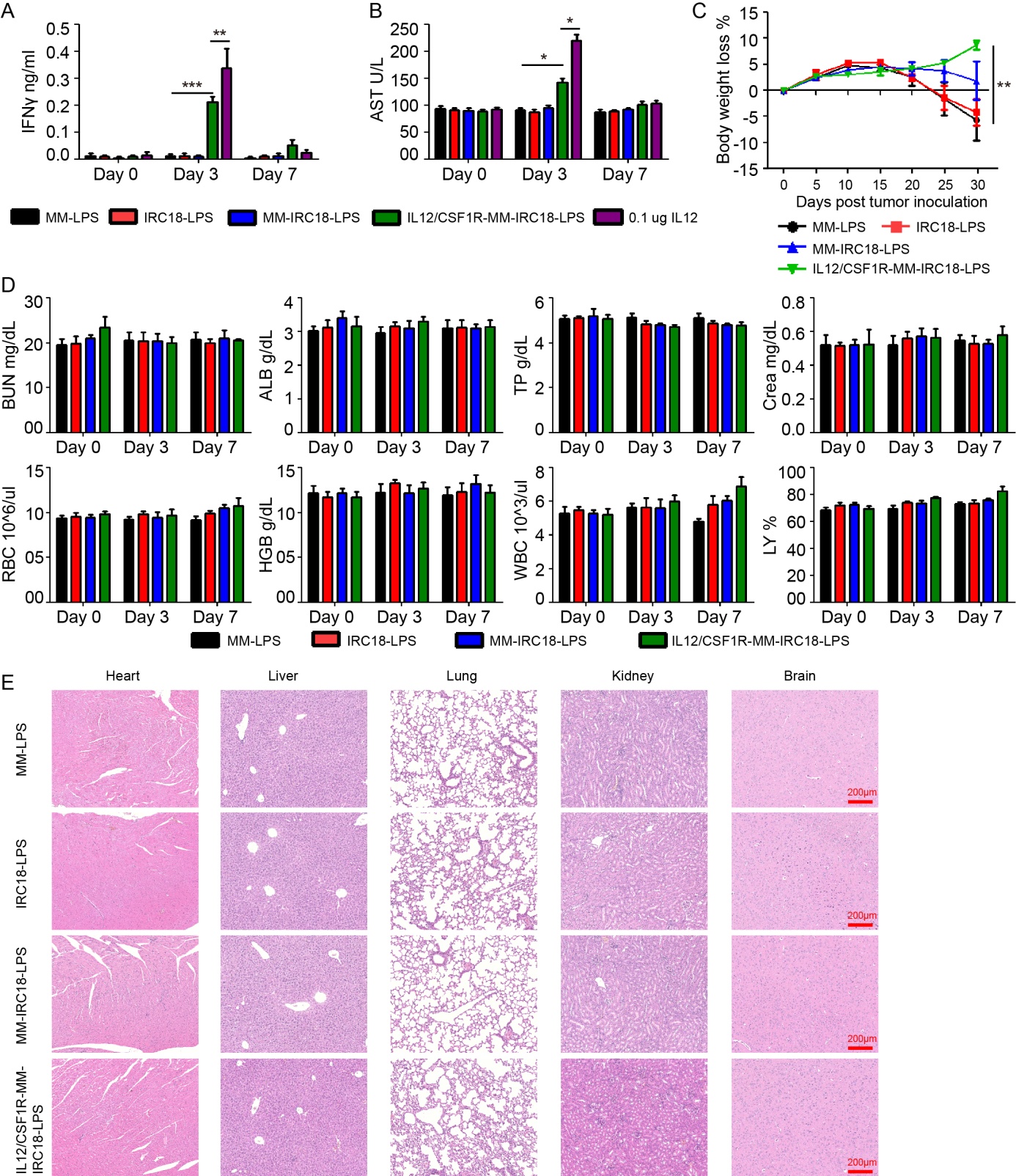


**Figure S9. (A-B),** IFNγ (A) and blood Aspartate transaminase (AST) (B) concentration at day 0, 3, and 7 after C57BL/6 mice were treated with MM-LPS, IRC18-LPS, MM-IRC18-LPS, IL12/CSF1R-MM-IRC18-LPS, and 0.1 μg recombinant mouse IL12 (n=3 for each group). **(C)** GBM-bearing mice were administrated with samples at day 7, 14, and 21 after GBM inoculation. 12 hours post-administration, the mice brains were exposed to the laser irradiation (808 nm, 1.0 W cm^-2^, 10 min). The mice were weighed every two days. (3-6 mice per group). **(D)** Blood biochemistry at day 0, 3, and 7 after treatment (n=3). Blood urea nitrogen (BUN), Albumin (ALB), Total Protein (TP), Creatinine (Crea), Hemoglobin (HGB), Red blood cells (RBC), White blood cells (WBC), and percent of lymphocytes (LY %). **(E)** Hematoxylin & Eosin staining of heart, liver, lung, kidney, and brain from mice at day 7 after indicated treatment. Data are representative of three independent experiments (mean ± SEM ). *p < 0.05, **p < 0.01, ***p < 0.001 by Student’s t test.


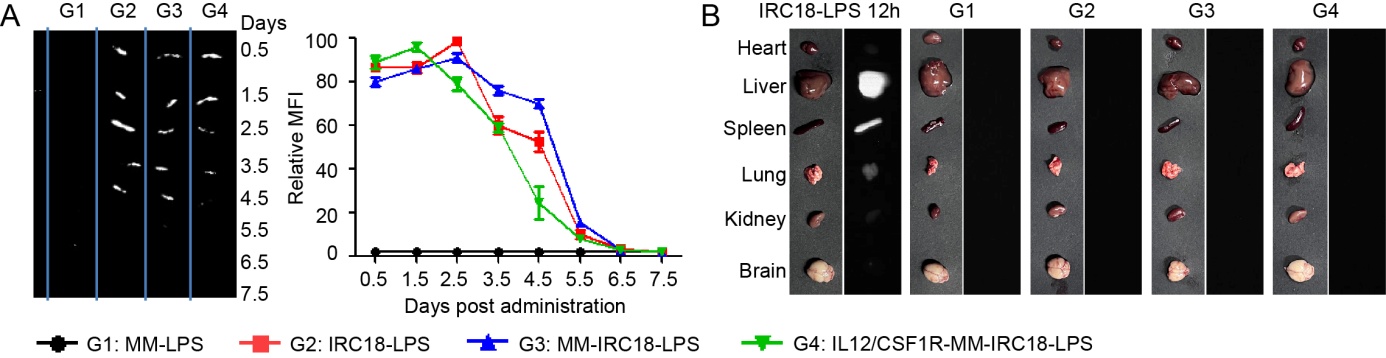


**Figure S10. (A)** NIR-II fluorescence imaging (808 nm, 40mW/cm^2^, 1000LP, 1000 ms) of the mouse faeces collected at indicated time points after a single tail vein injection. Quantitative results of NIR-II fluorescence intensity of the faeces (n=3). **(B)** NIR-II fluorescence imaging (808 nm, 40mW/cm^2^, 1000LP, 400 ms) of the main organs (heart, liver, spleen, lung, kidney, and brain) collected on day 7.5 after a single tail vein injection. IRC18-LPS 12h indicates the organs collected 12 hours after a single tail vein injection of IRC18-LPS, serving as a positive control for imaging. Data are representative of three independent experiments (mean ± SEM ). G1, Group 1, MM-LPS; G2, Group 2, IRC18-LPS; G3, Group 3, MM-IRC18-LPS; G4, Group 4, IL12/CSF1R-MM-IRC18-LPS.

**References**

[1] Rao L, Zhao SK, Wen C, Tian R, Lin L, Cai B, et al. Activating Macrophage-Mediated Cancer Immunotherapy by Genetically Edited Nanoparticles. Advanced materials. 2020;32:e2004853.

[2] Piffoux M, Silva AKA, Wilhelm C, Gazeau F, Tareste D. Modification of Extracellular Vesicles by Fusion with Liposomes for the Design of Personalized Biogenic Drug Delivery Systems. ACS nano. 2018;12:6830-42.

[3] Liu Y, Li M, Gu J, Huang H, Xie H, Yu C, et al. Engineering of exosome-liposome hybrid-based theranostic nanomedicines for NIR-II fluorescence imaging-guided and targeted NIR-II photothermal therapy of subcutaneous glioblastoma. Colloids and surfaces B, Biointerfaces. 2024;245:114258.

[4] Antaris AL, Chen H, Cheng K, Sun Y, Hong G, Qu C, et al. A small-molecule dye for NIR-II imaging. Nature materials. 2016;15:235-42.

[5] Yuan Y, Feng Z, Li S, Huang Z, Wan Y, Cao C, et al. Molecular Programming of NIR‐IIb‐Emissive Semiconducting Small Molecules for In Vivo High‐Contrast Bioimaging Beyond 1500 nm. Advanced Materials. 2022;34.

[6] Jiang Y, Huang J, Xu C, Pu K. Activatable polymer nanoagonist for second near-infrared photothermal immunotherapy of cancer. Nature communications. 2021;12:742.
